# The Impact of Resource Inequality on Cooperative Behavior in Social Dilemmas

**DOI:** 10.3390/bs15040519

**Published:** 2025-04-13

**Authors:** Jieyu Lv, Huan Wang, Wei Cai, Danli Yang, Yonghong Yu

**Affiliations:** 1 Department of Psychology, School of Sociology and Psychology, Central University of Finance and Economics, Beijing 100081, China; cjamor@163.com (H.W.); 2022212206@email.cufe.edu.cn (D.Y.); 2 Research Center for Quality of Life and Applied Psychology, School of Humanities and Management, Guangdong Medical University, Dongguan 523808, China; weicai@gdmu.edu.cn

**Keywords:** behavioral heterogeneity, social dilemma, fairness perception, group identity, resource inequality

## Abstract

Previous research has not yet identified the psychological mechanisms underlying the impact of resource inequality on cooperative behavior. To further explore this issue, this study used two single-factor experiments to investigate the influence of resource inequality on cooperative behavior in social dilemmas, focusing on the mediating role of fairness perception and group identity. The results showed that behavioral heterogeneity was higher under unequal conditions than that under equal conditions. In addition, in unequal groups, high-endowment players exhibited lower cooperation levels than low-endowment players. However, the mediating roles of fairness perception and group identity were not confirmed. This study highlights the complexity of resource inequality’s influence on group cooperation and offers new directions for future research.

## 1. Introduction

We often face the choice between pursuing our own best interests and sacrificing for the collective good in our daily life (Deutchman et al., 2022; van Lange et al., 2013). These situations are known as “social dilemmas”. Social dilemmas can be categorized into symmetric and asymmetric types based on the equality of certain dimensions or factors (Liu & Hao, 2015). In asymmetric social dilemmas, the symmetry is reflected in factors such as initial resources, opportunities, benefits, information and status (Anderson et al., 2008; Cherry et al., 2005). This study examines the influence of resource inequality on cooperative behavior at both group and individual levels by manipulating differences in the initial endowments of public goods.

Previous studies have found that resource inequality negatively impacts group-level cooperation, suggesting that unequal resource distribution leads to a reduction in the provision of public goods (Aquino et al., 2016; Cherry et al., 2005; Hargreaves Heap et al., 2016). For example, Rapoport and Suleiman (1993) observed that groups with unequal resource distribution contributed less to the public goods than those with equal resource distribution. Similarly, Cherry et al. (2005) found that groups with equal resource distribution contributed more to the public goods than those with unequal resource distribution, regardless of whether the initial resources were acquired through effort or by chance. These findings suggest that the uneven distribution of resources within a group leads to differentiation within the group, which stimulates the self-interest of individuals and makes them less inclined to contribute, thus weakening the level of group cooperation. Based on these observations, we propose our Research Hypothesis 1: *Resource inequality is likely to reduce group cooperative behavior and increase individual behavioral heterogeneity within the group.*

Resource inequality can lead to behavioral heterogeneity among group members. Some studies have shown that the degree of individual contribution to the collective good is related to the amount of resources possessed (Grabka & Goebel, 2018). While some studies support the idea that “the more you have, the more you give”—with wealthier individuals paying more attention to group interests and contributing more to promote social equity and narrow the gap with others (Joel, 2023; van Dijk & Wilke, 1995)—a great body of research supports the opposite view: “the more you have, the less you give” view. This suggests that individuals with more resources tend to exhibit less cooperative behavior (Piff et al., 2010). The amount of resources influences the strength or weakness of one’s social status. With increased material resources, educational opportunities, and less pressure, individuals in more powerful positions tend to be more independent and self-centered, leading to lower contributions to public goods (Hargreaves Heap et al., 2016; Ramalingam & Stoddard, 2024) and lower prosociality (Hauser et al., 2019; Piff et al., 2010). Therefore, we propose our Research Hypothesis 2: *In a group with resource inequality, high-endowment players exhibit lower contribution rates than low-endowment players*.

Social exchange theory (Homans, 1958) and the group engagement model (Tyler & Blader, 2003) are theoretical frameworks that explain cooperative behavior based on resource exchange and group identity. Social exchange theory focuses on the dynamic process of resource exchange, emphasizing the role of resource allocation in shaping individual behavior. In contrast, the group engagement model centers on group identity, asserting that a strong sense of belonging to the group is essential to promoting cooperative behavior. Building on the role of fairness perception in resource exchange and the structure of group identity in the group engagement model, this study aims to explore the psychological mechanism through which fairness perception and group identity impact cooperative behavior in the context of resource inequality.In groups with unequal resources, resource disparity triggers comparative thinking among members and evaluates the fairness of resource allocation, thus generating fairness perception (Jasso et al., 2016). Fairness perception is a key factor influencing individuals’ contribution to group public resources, and individuals who perceive unfair resource distribution are less willing to cooperate in public goods issues (Colquitt & Zipay, 2015; Hart & Piff, 2018; Ramalingam & Stoddard, 2024; Zhang, 2017; Zhu et al., 2022). Therefore, we propose our Research Hypothesis 3: *Fairness perception plays a mediating role between resource inequality and individual cooperative behavior.* Moreover, group identity refers to an individual’s sense of belonging and identification with the group. The group engagement model posits that individuals’ identification with the group determines their willingness to participate in group activities. When members within a group receive unequal amounts of initial resources, this disparity may affect individuals’ sense of identity with the group and, consequently, their cooperative behavior (Aksoy, 2019). Therefore, we propose our Research Hypothesis 4: *Individuals’ sense of group identity plays a mediating role between resource inequality and individual cooperative behavior.*

In order to examine the research hypotheses, two experiments were designed to explore the influence of resource inequality on cooperative behavior in contributing to public goods and its underlying psychological mechanism. The goal of Experiment 1 was to examine the direct impact of resource inequality on cooperative behavior in contributing to public goods. We adopted the public goods paradigm as in previous studies (Cherry et al., 2005; Lv et al., 2025b; Nockur et al., 2021). The experiment involved two conditions: the Equal Resources 20-20 condition, where the total resources within the group were 40 tokens and each participant received 20 tokens, and the Unequal Resources 40-20 condition, where the total resources within the group were 60 tokens in Experiment 1. In the latter condition, one participant received 40 tokens (high endowment) and another received 20 tokens (low endowment), ensuring that the initial low endowment was identical to that of the Equal Resources 20-20 condition. This design allowed us to examine how differences in initial endowments affect cooperative behavior, although it did not directly compare groups with identical total resources but different individual endowments. To address this limitation, Experiment 2 introduced a new inequality condition: the Unequal Resources 30-10 condition, where the total resources in the group were again 40 tokens, but one participant received 30 tokens (high endowment), and the other received 10 tokens (low endowment). This condition was designed to control for the total amount of group resources while varying the contributions. Experiment 2 also added the measurement of group identity to investigate whether the resource inequality’s effect on cooperative behavior would be influenced by controlling for the total group resources, further illuminating the psychological mechanisms underlying these effects. The main purpose of Experiment 2 was to test the mediating role of group identity and rule out the potential influence of varying total resources from Experiment 1. Experiment 1 relied on tangible tokens and traditional paper–pencil methods for data collection, and the approach proved to be time-consuming and costly. To improve efficiency, Experiment 2 was conducted on the oTree platform (Chen et al., 2016; Lv et al., 2025a), which significantly streamlined the data collection process. For those interested in a deeper exploration of our methods and findings, we have made our experimental programs, raw datasets, and the R code used for data analysis available on the Open Science Framework at https://osf.io/j3v2t/ (accessed on 9 January 2025).

## 2. Experiment 1: Effects of Resource Inequality on Cooperation in Social Dilemmas

### 2.1. Method

#### 2.1.1. Participants

Before data collection, we conducted a statistical power analysis to infer the requirement of sample size to reach 1−β=0.8 by the pwr package (version 1.3-0) in R (version 4.4.3). We set effect size d=0.8 and significance level α=0.05, and we calculated that in each condition, 26 participants were needed. A total of 86 participants were recruited to attend this experiment via fliers and posters ($M_{\mathrm{age}}\;=\;19.40,\;SD_{\mathrm{age}}\;=\;1.40$). There were 64 females and 22 males. All participants signed the consent inform and read the study notice. Participants’ payment was positively correlated with their payoff in the public goods game in the experiment. This study obtained the research approval of the Research Committee of Central University of Finance and Economics (IRB20240520001). The experimental protocol is described in the Supplementary Materials.

#### 2.1.2. Design

**Figure 1:**
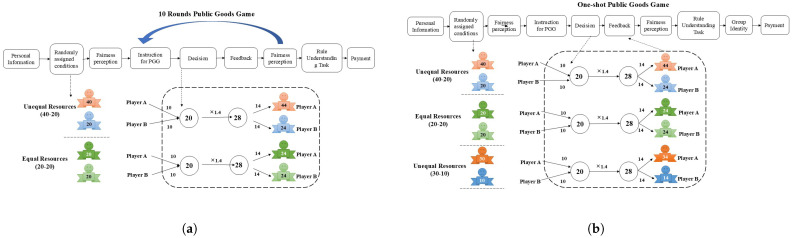
Experimental flow for Experiment 1 and Experiment 2. (**a**) Experimental flow for Experiment 1. (**b**) Experimental flow for Experiment 2.

Experiment 1 adopted a one-factor, two-level between-subject design (resource inequality: Unequal Resources 40-20/Equal Resources 20-20), as shown in Figure 1a. Resource inequality was the independent variable with two levels: Unequal Resources 40-20 and Equal Resources 20-20 conditions. Players A and B under the Unequal Resources 40-20 condition were assigned 40 and 20 tokens, respectively; Player A and Player B under the Equal Resources 20-20 condition were assigned 20 tokens each. Cooperation, operationalized as the contribution rate, served as the dependent variable in this study. The contribution rate was calculated as the proportion of an individual’s contribution relative to their total available resources within the group context. Other variables included fairness perception.

#### 2.1.3. Materials

##### A Two-Player Public Goods Game

As illustrated in Figure 1a, we implemented a 10-round, two-player linear public goods game. The experiment utilized five distinct colored tokens, i.e., red, blue, light green, dark green and yellow, to symbolize the tokens used within the game. At the beginning of each round, under the Unequal Resources 40-20 condition, Player A was assigned 40 tokens (red), while Player B was assigned 20 tokens (blue); Player A and Player B under the Equal Resources 20-20 condition were assigned 20 tokens each (light green and dark green, respectively). Moreover, the two players knew the number of tokens of the other. Each player needed to decide the number of tokens they would transfer from their personal account to the public pool. The combined contributions from Player A and Player B to this pool would accrue an additional interest of 40%. Subsequently, the amassed tokens in this communal pool would be equally distributed between the two players. An integral component of the game dynamics was feedback. After every round, players received feedback detailing “their contribution”, “the other player’s contribution”, “their share of tokens from the public pool”, and “their accumulated tokens”. Such paradigm has been previously employed in Osman et al. (2018)’s study.

##### Rule Understanding Task

We measured whether participants understood the rule of the public goods game by asking them four questions, such as “*If you contribute 20, Player 2 contributes 20, how many tokens you will receive in the end?*”.

##### Fairness Perception

We adopted a 7-point Likert scale to measure fairness perception, including cognition and emotion parts. The cognition part included two items with rating 1 *(very dissatisfied)* to 7 *(very satisfied)* or 1 *(very unfair)* to 7 *(very fair)*. The question was the following: “How fair do you think the assignment of initial tokens between you and the other participant is?” The emotion part included seven items with rating 1 *(strongly disagree)* to 7 *(strongly agree)*. The question was the following: “*How well does this word describe your current emotional state?*”. We measured fairness perception twice, once before and once after the public goods game. We asked participants to rate seven adjectives of emotions, including calm, pleasant, angry, excited, guilty, disappointed, and sad. We measured satisfaction and fairness perception towards the initial endowment deal between the two players in the first fairness perception task. We added two more questions to make a comparison with the first fairness perception task (“*Are you satisfied with your contribution of public goods game?*” and “*Are you satisfied with the other player’s contribution of public goods games?*”) in the second fairness perception task.

##### Debriefing Questions

In the end, we interviewed participants by asking them several questions, including some regarding the decision strategy used in the public goods game.

#### 2.1.4. Procedure

As illustrated in Figure 1a, all participants were asked to complete a face-to-face paper–pencil test in pairs. First, the participants read the instructions. After confirming their understanding, they signed an informed consent form and a notification outlining the study’s content. They also provided personal information such as age and gender. Next, each participant was assigned a certain number of tokens. Within the same group, the number of tokens each participant received was disclosed. Once the token numbers were disclosed, the participants were asked to complete the first fairness perception scales, which assessed their perception of the fairness of the initial resource allocation between the two players. The participants then played ten rounds of the public goods game. During each round, the participants placed their tokens into their individual choice boxes and handed them to the experimenter once both participants had made their decisions. The process for each round was the same: the participants’ token number remained unchanged, and they independently decided how many tokens to contribute to the public pool. After each round, participants received feedback on the results, which included “their own contribution”, “the other participants’ contribution”, “the total number of tokens in the current round” and “the cumulative total of tokens”. After completing all ten rounds, participants were asked to fill out the fairness perception scales again, including their satisfaction with their own performance and the other player’s. Participants then completed a rule understanding test. They answered a series of questions to verify whether they understood the purpose of the experiment and the strategy they had used in the task. Finally, participants were paid in cash based on the total number of tokens earned across the ten rounds at the exchange rate of 25 tokens being equal to CNY 1. On average, participants received between CNY 5.6 and CNY 13.6 for participating in our experiments.

### 2.2. Results

#### 2.2.1. Group Cooperation Level Under Unequal Resources and Equal Resources Conditions

We conduced an independent *t*-test with resource inequality as the independent variable and the average contribution in the public goods game across 10 rounds as the dependent variable. The results show that there was no significant difference in the average contribution across 10 rounds between the Unequal Resources 40-20 condition (M=33.01, SD=13.59) and the Equal Resource 20-20 condition (M=29.46, SD=7.25) (t(30.22)=−1.06, p=0.30, Cohen’sd=−0.32).

The group relative contribution rate was the proportion of contribution out of the total endowments for the whole group. We conducted an independent *t*-test with resource inequality as the independent variable and the average contribution rate across 10 rounds for the group as the dependent variable. The results show that the mean contribution rate of the group under the Unequal Resources 40-20 condition (M=0.55, SD=0.23) was significantly lower than under the Equal Resources 20-20 condition (M=0.74, SD=0.18) (t(38.30)=2.97, p=0.01, Cohen’sd=0.91).

**Figure 2:**
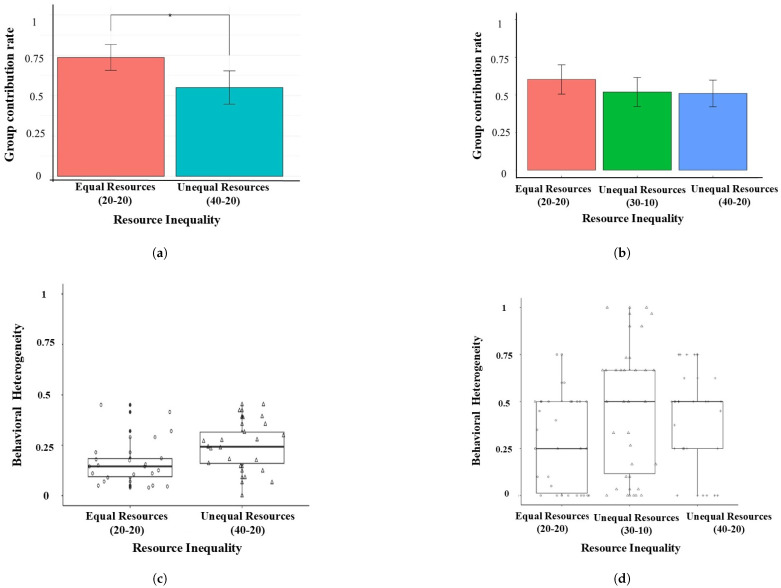
Group cooperation level and behavioral heterogeneity under the Unequal and Equal Resources conditions (left, Experiment 1; right, Experiment 2). (**a**) Bar chart for Experiment 1. (**b**) Bar chart for Experiment 2. (**c**) Box plot for Experiment 1. (**d**) Box plot for Experiment 2. * *p* < 0.05.

Behavioral heterogeneity was measured by the difference in the absolute contribution rate of the players within a group. We conducted an independent *t*-test with resource inequality as the independent variable and behavioral heterogeneity as the dependent variable. The results show that the behavioral heterogeneity under the Unequal Resources 40-20 condition (M=0.24, SD=0.12) was significantly higher than that under the Equal Resources 20-20 condition (M=0.16, SD=0.11) (t(40.43)=−2.17, p=0.04, Cohen’sd=−0.66), as illustrated in Figure 2a.

#### 2.2.2. Cooperation Among Players Under Unequal Resources Condition

**Figure 3:**
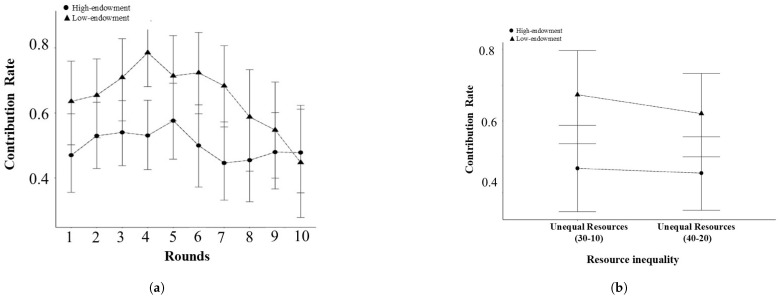
Contribution rate across varying levels of resource inequality under Unequal Resources conditions: (**a**) Experiment 1. (**b**) Experiment 2.

Table 1 shows the absolute contribution and the contribution rates of individuals under unequal conditions. We conducted an independent sample *t*-test with resource inequality as the independent variable and the average contribution rate under the Unequal Resources 40-20 condition as the dependent variable. The results show that high-endowment players exhibited significantly lower average contribution rates across the 10 rounds ($M_{\mathrm{high}}=0.50,\;SD_{\mathrm{high}}=0.24$) than players with less resources under the Unequal Resources 40-20 condition ($M_{\mathrm{low}}=0.65,\;SD_{\mathrm{low}}=0.23$) (t(39.98)=−2.03, p<0.05, Cohen’sd=−0.63), as illustrated in Figure 3a.

#### 2.2.3. Mediating Effect: Fairness Perception

We conducted an independent *t*-test with resource inequality as the independent variable and the first fairness perception score as the dependent variable. The results show that the overall fairness perception under the Unequal Resources 40-20 condition (M=3.62, SD=1.29) was lower than that under the Equal Resources 20-20 condition (M=5.09, SD=1.36) (t(83.99)=5.15, p=0.001, Cohen’sd=1.11). Further analysis distinguished individuals with different amounts of resources under the Unequal Resources 40-20 condition. The results show that high-endowment players showed a slightly higher fairness perception (M=3.71, SD=1.01) than low-endowment players (M=3.52, SD=1.54).

We conducted a mediation analysis with the R “*mediation*” package by setting fairness perception as the mediation variable, and the sampling frequency was set to 5000. We conducted a mediation analysis with resource inequality as the independent variable, the first fairness perception as the mediation variable and the average contribution rate across the 10 rounds as the dependent variable. The results show that the mediation effect of fairness perception was not significant (ACME=0.05, 95%CI[−0.004,0.11], p=0.07) but the direct effect of resource inequality on the contribution rate was significant (ADE=−0.20, 95%CI[−0.31,−0.10], p<0.001). The total effect of resource inequality on the contribution rate was significant (TotalEffect=−0.16, 95%CI[−0.26,−0.06], p<0.001).

In a deeper analysis, we incorporated the role (Player A/Player B) into the model as a moderating variable to explore whether it significantly moderated the mediating effect of perceived fairness on the effect of resource inequality on the average contribution rate across the 10 rounds. However, the results show that the mediating effect was not significantly moderated by the role (${\mathrm{ACME}}_{A}=0.05,\;95\%\mathrm{CI}\left[-0.001,0.10\right],\;p=0.06$; ${\mathrm{ACME}}_{B}=0.04,\;95\%\mathrm{CI}\left[-0.002,0.10\right],\;p=0.06$).

## 3. Experiment 2

### 3.1. Method

#### 3.1.1. Participants

Before data collection, we conducted a statistical power analysis to infer the requirement of sample size to reach a statistic power of (1−β)=0.8 by the pwr package in R. We set effect size f=0.25 and significance level α=0.05, and we calculated that in each condition, 53 participants were need. A total of 186 participants were recruited to attend this experiment via fliers and posters. Eight participants were not included in the data analysis due to part of the data being missing. Since the data were paired, some data were removed for this reason. Thus, the valid participants in this study for data analysis were 178 ($M_{\mathrm{age}}=18.94,\;SD_{\mathrm{age}}=0.83$), with 100 females and 78 males.

#### 3.1.2. Design

Experiment 2 adopted a one-factor, three-level between-subject design (resource inequality: Unequal Resources 30-10/Unequal Resources 40-20/Equal Resources 20-20), as shown in Figure 1b. Resource inequality was the independent variable with three levels: Unequal Resources 30-10, Unequal Resources 40-20 and Equal Resources 20-20 conditions. The Unequal Resources 30-10 condition comprised 40 tokens for the group, and Player A and Player B were assigned 30 and 10 tokens, respectively. The Unequal Resources 40-20 condition comprised 60 tokens for the group, and Player A and Player B were assigned 40 and 20 tokens, respectively. The Equal Resources 20-20 condition comprised 40 tokens for the group, and Player A and Player B were assigned 20 tokens each. The number of tokens for the group under the Unequal Resources 30-10 condition was identical to that of the Equal Resources 20-20 condition. The dependent variable in Experiment 2 was identical to the dependent variable in Experiment 1. Other variables included fairness perception and group identity, where we added group identity compared with Experiment 1.

#### 3.1.3. Materials

##### Public Goods Game

Experiment 2 employed the same public goods game with one shot instead of the ten rounds of Experiment 1. Moreover, we presented graphic instructions for Experiment 2, rather than the textual instructions in Experiment 1, to enhance participants’ understanding.

##### Rule Understanding Task

The rule understanding task was identical to Experiment 1.

##### Fairness Perception Tasks

The fairness perception tasks were identical to Experiment 1.

##### Group Identity Measures

Participants were required to fill in the identity scale after the public goods game, with seven scales from 1 *(disagree with)* to 7 *(agree with)*.The question was the following: “*To what extent do you agree with that you have built a team with the other player?*”

#### 3.1.4. Procedure

Figure 1b presents the procedure of Experiment 2. Groups of six participants arrived at the laboratory, opened a web browser, logged in using the provided link, and entered their tag numbers to begin the experiment. The participants entered some personal information and were then randomly assigned to one of the experimental conditions: Unequal Resources 30-10, Unequal Resources 40-20 or Equal Resources 20-20. Following this, they completed the first fairness perception task. Next, they read the instructions for the public goods game, made their decisions, and received feedback. Subsequently, they filled in the second fairness perception task and the rule understanding task. Finally, they responded to the group identity question and got paid based on the performance in the public goods game at an exchange rate of 2.5 tokens being equal to CNY 1. On average, participants received between CNY 5.6 and CNY 13.6 for participating in our experiments.

### 3.2. Results

#### 3.2.1. Group Cooperation Level Under Unequal and Equal Resources Conditions

We conducted a one-way ANOVA with resource inequality as the independent variable and the average contribution in the public goods game as the dependent variable. The results show that there was a significant difference in group contribution at different levels of resource inequality ($F\left(2,86\right)=5.42,\;p<0.01,\;\eta_{p}^{2}=0.11$). A post hoc test revealed that the group contribution under the Unequal Resources 40-20 condition (M=30.59, SD=13.75) was significantly higher than under the Equal Resources 20-20 condition (M=24.10, SD=10.34) (p<0.05) and the Unequal Resources 30-10 condition (M=20.77, SD=10.25) (p<0.01).

We conducted a one-way ANOVA with resource inequality as the independent variable and the group contribution rate as the dependent variable. The results show that there was no significant difference among different types of resource inequality, the Equal Resource 20-20 condition (n=30, M=0.60, SD=0.26), the Unequal Resources 30-10 condition (n=30, M=0.52, SD=0.26) and the Unequal Resource 40-20 condition (n=29, M=0.51, SD=0.23) (F(2,86)=1.23, $p=0.30,\;\eta_{p}^{2}=0.03$).

We conducted a one-way ANOVA with resource inequality as the independent variable and the absolute value of the contribution rate difference among players within a group. The results of the *F*-test show that there were significant differences in behavioral heterogeneity under the various resource inequality conditions ($F\left(2,86\right)=2.77,\;p=0.068,\;\eta_{p}^{2}=0.06$). Post hoc tests revealed that the behavioral heterogeneity under the Unequal Resources 30-10 condition (M=0.45, SD=0.33) was significantly higher than that under the Equal Resources 20-20 condition (M=0.28, SD=0.23) (p=0.026). There was no statistical difference in the behavioral heterogeneity under the Unequal Resources 30-10 and the Unequal Resources 40-20 conditions (p=0.60), and the Unequal Resources 40-20 condition and the Equal Resources 20-20 condition (p=0.09).

#### 3.2.2. Cooperation Among Players Under Unequal Resources Conditions

We conducted a one-way ANOVA with resource inequality as the independent variable and the individual contribution rates as the dependent variables. The results show that there was no significant difference among the three conditions of resource inequality (F(2,175)=0.54, $p=0.58,\;\eta_{p}^{2}=0.01$).

We conducted a two-way ANOVA with resource inequality (Unequal Resources 40-20/Unequal Resources 30-10) and role (Player A/Player B) as the independent variables andthe individual contribution rate as the dependent variable. The results show that the main effect of the role was significant ($F\left(1,116\right)=5.50,\;p=0.02,\;\eta_{p}^{2}=0.05$).The individual contribution rate of high-endowment players ($M_{\mathrm{high}}=0.46,\;SD_{\mathrm{high}}=0.31$) was significantly lower than that of low-endowment players ($M_{\mathrm{low}}=0.65,\;SD_{\mathrm{low}}=0.36$) (p=0.003). The main effect of the unequal condition was not significant (F(1,116)=0.0001, p=0.99). The interaction between the unequal condition and role was not significant (F(1,116)=0.187, p=0.66).

#### 3.2.3. Mediating Effect: Fairness Perception and Group Identity

The mediating effect test was conducted with the same tools as Experiment 1, and the multiple sampling times were set to 5000 times. Model 1 was set with resource inequality as the independent variable, the first fairness perception as the mediation variable, and the contribution rate as the dependent variable to conduct a mediation analysis. The results show that the mediation effect of fairness perception was not significant (ACME=0.08, 95%CI[−0.03,0.19], p=0.14), the direct effect of resource inequality on the contribution rate was not significant (ADE=−0.09, 95%CI[−0.26,0.07], p=0.27) and the total effect of resource inequality on the contribution rate was not significant (TotalEffect=−0.02, 95%CI[−0.14,0.11], p=0.81). The player type was further included in Model 1 as a moderating variable for analysis. The results show that the player type does not significantly regulate the mediating effect of fairness perception on the contribution rate of resource inequality (${\mathrm{ACME}}_{A}=0.08,\;95\%\mathrm{CI}\left[-0.01,0.18\right],\;p=0.10;\;{\mathrm{ACME}}_{B}=0.08$, 95%CI[−0.02,0.18], p=0.12). Model 2 was set with resource inequality as the independent variable, group identity as the mediation variable and the contribution rate as the dependent variable to conduct a mediation analysis. The results show that the mediation effect of group identity was not significant (ACME=0.02, 95%CI[−0.01,0.06], p=0.16), the direct effect of resource inequality on the contribution rate was not significant (ADE=−0.04, 95%CI[−0.17,0.10], p=0.56) and the total effect of resource inequality on the contribution rate was not significant (TotalEffect=−0.02, 95%CI−0.16,0.11], p=0.75). The player type was further included in Model 2 as a moderating variable for analysis, and the results show that the player type does not significantly regulate the mediating effect of group identity on the contribution rate of resource inequality (${\mathrm{ACME}}_{A}=0.02,\;95\%\mathrm{CI}\left[-0.004,0.05\right]$, $p=0.13;\;{\mathrm{ACME}}_{B}=0.02,\;95\%\mathrm{CI}\left[-0.004,0.05\right],\;p=0.11$).

**Figure 4:**
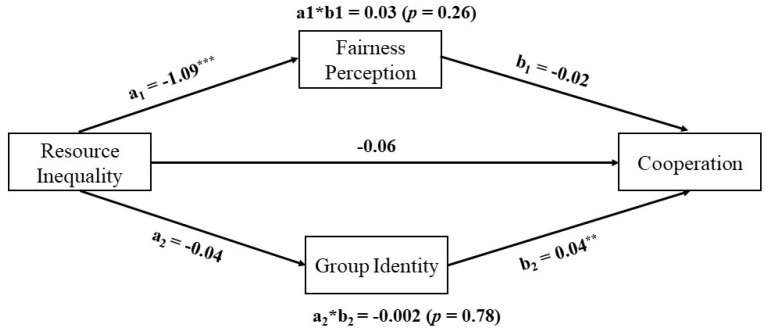
Parallel model for fairness perception and group identity (* *p* < 0.05, ** *p* < 0.01, *** *p* < 0.001).

In order to explore the influence of the possible interaction between fairness perception and group identity on the research results, we adopted R’s lavaan package (version 0.6-19) (Rosseel, 2012) for parallel mediation model analysis.This allocation takes the resource inequality conditions as the independent variables (Unequal Resources 30-10, Unequal Resources 40-20 and Equal Resources 20-20), fairness perception and group identity as the mediating variables and the individual contribution rate as the dependent variable.The results are shown below in Figure 4. The analysis results show that resource inequality did not directly impact the individual contribution rate (Estimate=−0.06, p=0.11). The mediating path coefficients through fairness perception were also not significant (Estimate=0.03, p=0.26), nor were they significant through group identity (Estimate=−0.002, p=0.78). Furthermore, the results of the mediation effect analysis of the double-mediation model were consistent with the results of the single-mediation model, and all the mediation effects were not significant.

## 4. General Discussion

The current study explored the impact of resource inequality on cooperation in social dilemmas and the underlying psychological mechanisms. In Experiment 1, it was observed that resource inequality reduced the contribution rate at the group level. Specifically, the average contribution rate was lower under the Unequal Resources 40-20 condition compared with the Equal Resources 20-20 condition. This experiment also highlighted that behavioral heterogeneity was more pronounced under the unequal condition, with high-endowment players contributing less than their low-endowment players under the Unequal Resources 40-20 condition. Interestingly, no evidence suggests that fairness perception mediated the relationship between resource inequality and the contribution rate. In contrast, Experiment 2 found no significant differences in contribution rates across various conditions of resource inequality. However, it did reveal significant variability in the consistency of cooperative behavior within groups. Especially under unequal conditions, individual cooperative behavior inconsistency was significantly higher than that under the Equal Resources 20-20 condition. This suggests that resource inequality leads to more fragmented behavior within groups. Under both the Unequal Resources 40-20 and Unequal Resources 30-10 conditions of Experiment 2, the contribution rate of those with more resources was also lower than that of those with less resources. In addition, neither fairness perception nor group identity appear to mediate the effect of resource inequality on the individual contribution rate. While the results regarding group contribution rates vary between the experiments, both confirmed that behavioral heterogeneity was elevated under unequal conditions. High-endowment players exhibited lower cooperative behavior than low-endowment players under these conditions, partly supporting our Hypothesis 1, which proposed that resource inequality reduces group cooperation. However, this hypothesis was not fully supported. In addition, the consistent finding across both experiments that high-endowment players exhibited less cooperative behavior than low-endowment players supports Hypothesis 2. Yet, neither experiment provided evidence that fairness perception or group identity mediates the relationship between resource inequality and cooperative behavior, leaving Hypotheses 3 and 4 untested.

### 4.1. Effects of Resource Inequality on Group Cooperation

The results from Experiment 1 indicate no significant differences in group contributions between the Equal Resources 20-20 condition and the Unequal Resources 40-20 condition. Despite the substantial disparity in initial total resources, both groups contributed an equivalent amount. Conversely, Experiment 2 demonstrated that resource inequality significantly influences group contribution, particularly showing that the contributions under the Unequal Resources 40-20 condition were significantly higher than those in the other groups. The results from the two experiments present an inconsistency for the Unequal Resources 40-20 condition and the Equal Resources 20-20 condition. It was hypothesized that groups with greater resources might contribute more overall, yet the experimental results did not corroborate this assumption. Regarding the average contribution rate, Experiment 1 identified a difference between the Unequal Resources 40-20 and Equal Resources 20-20 conditions, which Experiment 2 did not replicate. Overall, the results align with previous research (Fung & Au, 2014), suggesting that the influence of resource inequality on group cooperative behavior is complex and dynamic, rather than straightforward. This effect may be modulated by various factors, such as the visibility of inequality and the kurtosis of asymmetry. The inconsistency between the two experiments may also suggest that the influence is unstable, potentially due to Experiment 1 being a multi-round task and Experiment 2 being a one-shot public goods game, where the interaction dynamics of the former could be affected by feedback on outcomes and other unaccounted factors.

To further investigate the impact of resource inequality on individual cooperative behavior, this study not only examined the magnitude of contributions within groups and the proportion of individual contributions to total group contributions but also explored behavioral heterogeneity in individual cooperative behavior within groups. The presence of resource inconsistency among individuals within a group can be influenced by various factors, such as the total amount of resources available to the group and whether interactions are conducted face to face. While this study did not arrive at a stable conclusion regarding the quantitative impact of resource inequality on individual cooperative behavior, it did identify notable variability in behavior within groups. Both Experiment 1 and Experiment 2 demonstrated that under unequal conditions, individual cooperative behavior exhibited greater behavioral heterogeneity. This insight suggests that cooperative behavior in unequal groups may be significantly affected by situational factors, enhancing our understanding of how resource inequality affects both collective and individual cooperative behavior. Future studies could delve deeper into how situational factors related to resource inequality influence cooperative behavioral heterogeneity.

### 4.2. Cooperation for Different Resource Players

Both experiments support the hypothesis that under conditions of resource inequality, high-endowment players contribute a significantly lower rate than low-endowment players, thereby confirming our Research Hypothesis 2. These results are consistent with previous studies (Hargreaves Heap et al., 2016; Li et al., 2013; Piff et al., 2010) which observed that wealthier individuals tend to cooperate less than their less affluent counterparts. The unequal distribution of resources results in distinct behaviors among individuals with varying levels of resources. In contexts of resource inequality, individuals are more aware of the disparities caused by the uneven distribution of resources. Before acting, they often assess the situation: “In this situation (situation recognition), for someone like me (identification), what should I do (action)?” (Weber et al., 2004). Additionally, resource inequality influences the selection of rules within groups. Similarly, high-endowment players often exhibit greater confidence due to their increased options and control, whereas low-endowment players, being more reliant on social connections, are generally more willing to cooperate. Consequently, low-endowment players contributed more to the collective good under unequal conditions, and their willingness to cooperate was significantly higher than that of high-endowment players.

### 4.3. Mediating Effect: Fairness Perception and Group Identity

The results of this study do not confirm Hypothesis 3; the anticipated mediating role of fairness perception and group identity between resource inequality and cooperative behavior was not observed. According to social exchange theory, individuals develop perceptions of fairness based on the distribution of resources. When individuals perceive resource allocation as unfair, their willingness to cooperate may decrease, viewing such cooperation as unjust or disadvantageous. Conversely, perceived fairness in resource distribution enhances their likelihood to cooperate. However, these findings contradict previous studies (Zhang, 2017), which indicated a positive correlation between fairness perception and the intention to cooperate in the public goods dilemma.

Experiment 2’s findings do not support Hypothesis 4, which proposed that group identity is the psychological mechanism influencing individual cooperative behavior in the context of resource inequality. The group engagement model suggests that individuals’ motivation to participate in group cooperation is largely driven by their identification with the group, which primarily stems from judgments about resources. When members receive varying initial endowments, they assess their resources relative to others’, fostering a sense of group identity. This identification then influences their cooperative behaviors within the group. As Hogg (2003) notes, intra-group comparisons prompt members to seek similarities within their group and differences with other groups. Members who share similar endowments perceive a greater sense of similarity and thus more strongly identify with their group. This heightened group identification encourages members to value group benefits more and engage in more cooperative behaviors. Additionally, groups characterized by resource equality tend to exhibit a higher cooperation rate than those with resource inequality (Cherry et al., 2005; Tyler & Blader, 2003; Wenzel, 2007).

### 4.4. Limitations and Future Studies

This study has several limitations. Firstly, while it confirms the complex effects of resource inequality on cooperative behavior in social dilemmas, it does not explore specific nonlinear models. Future studies should employ a more refined experimental design to accurately investigate the nonlinear effects of resource inequality on cooperative behavior.

Secondly, this study was the first to use behavioral heterogeneity within a group as a measure of group cooperation, but its applicability requires further examination. Most prior research (Fung & Au, 2014; Hauser et al., 2019; Sadrieh & Verbon, 2006; Zhu et al., 2022) focused on how resource inequality affects the quantity of individual cooperative behaviors, with less attention being given to the dispersion of cooperative behaviors within groups.

Thirdly, although we aimed to explore the potential mediating role of fairness perception and group identity between resource inequality and cooperative behavior, the experimental data did not support the mediating effect of these variables. Future research should more thoroughly investigate the psychological mechanism underlying the relationship between resource inequality and cooperative behavior, considering not only the quantity but also the variability of cooperative behaviors. In addition, our study suggests that classical theories may be limited in explaining the impact of resource inequality on cooperative behavior. Future research might also explore alternative psychological mechanisms, such as tolerance for inconsistency, dynamic variables like feedback and the influence of the query order as suggested by query theory (Johnson et al., 2007), to enhance our understanding of these complex dynamics.

## Figures and Tables

**Table 1:** Absolute contributions and contribution rates of individuals under Unequal Resources conditions in Experiments 1 and 2.

	Absolute Contribution M(SD): High Endowment	Absolute Contribution M(SD): Low Endowment	Contribution Rate M(SD): High Endowment	Contribution Rate M(SD): Low Endowment
Unequal Resources 40-20 condition in E1	20.03 (9.57)	12.98 (4.69)	0.50 (0.24)	0.65 (0.70)
Unequal Resources 30-10 condition in E2	14.03 (9.97)	6.73 (3.76)	0.47 (0.33)	0.67 (0.38)
Unequal Resources 40-20 condition in E2	18.17 (11.96)	12.41 (0.34)	0.45 (0.30)	0.62 (0.34)

## Data Availability

The original data presented in the study are openly available on the Open Science Framework (https://osf.io/j3v2t/, accessed on 9 January 2025).

## Supplementary Materials

The following supporting information can be downloaded at: https://www.mdpi.com/article/10.3390/bs15040519/s1.
