# Supplementary material for: The Impact of Resource Inequality on Cooperative Behavior in Social Dilemmas
Source: Behav Sci (Basel). 2025 Apr 13;15(4):519. doi: 10.3390/bs15040519 (PMC12024223; doi:10.3390/bs15040519)
Supplement: Supplementary file 1 [file behavsci-15-00519-s001.zip › behavsci-3445874-supplementary.pdf]

Supplemental Information for Inequality and Cooperation Paper

[anonymous for review reason]

July 27, 2024

Contents

1 Fairness Test **2**
1.1 Fairness Test 1 2
1.2 Fairness Test 2 4

2 Rule Understanding Task **6**

3 Experimental Instructions for Experiments 1 and 2 **8**
3.1 Experimental Instructions for Experiment 1 8
3.1.1 Unequal 4020 condition 8
3.1.2 Equal 2020 condition 9
3.1.3 Equal2020 condition 21
3.2 Experimental Instructions for Experiment 2 21
3.2.1 Unequal3010 condition 21
3.2.2 Unequal4020 condition 35
3.2.3 Equal 2020 condition 36

1 Fairness Test

1.1 Fairness Test 1

Participant No.: ___________

Please tick the number that best matches your current state during the experiment.

1. How well does this word describe your current emotional state? Calm.

1	2	3	4	5	6	7
Strongly Disagree	Disagree	Somewhat Disagree	Neutral	Somewhat Agree	Agree	Strongly Agree

2. How well does this word describe your current emotional state? Happy.

1	2	3	4	5	6	7
Strongly Disagree	Disagree	Somewhat Disagree	Neutral	Somewhat Agree	Agree	Strongly Agree

3. How well does this word describe your current emotional state? Anger

1	2	3	4	5	6	7
Strongly Disagree	Disagree	Somewhat Disagree	Neutral	Somewhat Agree	Agree	Strongly Agree

4. How well does this word describe your current emotional state? Excitement.

1	2	3	4	5	6	7
Strongly Disagree	Disagree	Somewhat Disagree	Neutral	Somewhat Agree	Agree	Strongly Agree

5. How well does this word describe your current emotional state? Guilty.

1	2	3	4	5	6	7
Strongly Disagree	Disagree	Somewhat Disagree	Neutral	Somewhat Agree	Agree	Strongly Agree

6. How well does this word describe your current emotional state? Dissatisfied.

1	2	3	4	5	6	7
Strongly Disagree	Disagree	Somewhat Disagree	Neutral	Somewhat Agree	Agree	Strongly Agree

7. How well does this word describe your current emotional state? Sad

1	2	3	4	5	6	7
Strongly Disagree	Disagree	Somewhat Disagree	Neutral	Somewhat Agree	Agree	Strongly Agree

8. To what extent do you satisfied with the assignment of initial tokens between you and the other participant is?

1	2	3		5	6	7
Very Dissatisfied	Dissatisfied	Somewhat Dissatisfied	Neutral	Somewhat Satisfied	Satisfied	Very Satisfied

9. How fair do you think the assignment of initial tokens between you and the other participant is?

1	2	3		5	6	7
Very Unfair	Unfair	Somewhat Unfair	Neutral	Somewhat Fair	Fair	Very Fair

1.2 Fairness Test 2

Participant No.:________________

Please tick the number that best matches your current state during the experiment.

1. How well does this word describe your current emotional state? Calm.

1	2	3	4	5	6	7
Strongly Disagree	Disagree	Somewhat Disagree	Neutral	Somewhat Agree	Agree	Strongly Agree

2. How well does this word describe your current emotional state? Happy.

1	2	3	4	5	6	7
Strongly Disagree	Disagree	Somewhat Disagree	Neutral	Somewhat Agree	Agree	Strongly Agree

3. How well does this word describe your current emotional state? Anger

1	2	3	4	5	6	7
Strongly Disagree	Disagree	Somewhat Disagree	Neutral	Somewhat Agree	Agree	Strongly Agree

4. How well does this word describe your current emotional state? Excitement.

1	2	3	4	5	6	7
Strongly Disagree	Disagree	Somewhat Disagree	Neutral	Somewhat Agree	Agree	Strongly Agree

5. How well does this word describe your current emotional state? Guilty.

1	2	3	4	5	6	7
Strongly Disagree	Disagree	Somewhat Disagree	Neutral	Somewhat Agree	Agree	Strongly Agree

6. How well does this word describe your current emotional state? Dissatisfied.

1	2	3	4	5	6	7
Strongly Disagree	Disagree	Somewhat Disagree	Neutral	Somewhat Agree	Agree	Strongly Agree

7. How well does this word describe your current emotional state? Sad

1	2	3	4	5	6	7
Strongly Disagree	Disagree	Somewhat Disagree	Neutral	Somewhat Agree	Agree	Strongly Agree

8. To what extent do you satisfy with the assignment of initial tokens between you and the other participant is?

1	2	3		5	6	7
Very Dissatisfied	Dissatisfied	Somewhat Dissatisfied	Neutral	Somewhat Satisfied	Satisfied	Very Satisfied

9. How fair do you think the assignment of initial tokens between you and the other participant is?

1	2	3		5	6	7
Very Unfair	Unfair	Somewhat Unfair	Neutral	Somewhat Fair	Fair	Very Fair

10. To what extent do you satisfy with your own contribution during the games?

1	2	3		5	6	7
Very Dissatisfied	Dissatisfied	Somewhat Dissatisfied	Neutral	Somewhat Satisfied	Satisfied	Very Satisfied

11. To what extent do you satisfy with the other participants' contribution during the games?

1	2	3	4	5	6	7
Very Dissatisfied	Dissatisfied	Somewhat Dissatisfied	Neutral	Somewhat Satisfied	Satisfied	Very Satisfied

2 Rule Understanding Task

Rule Understanding Test

Let's say you are player 1 and you and Player 2 are each assigned with 20 tokens. Both of you are required to contribute some, all, or none of your tokens to a public fund (on a scale of 0 to 20). The tokens collected by the public fund will receive an additional 40% of the proceeds, and the tokens in the public fund will be divided equally between you.

Therefore, the number of tokens you have in a round of games will be calculated as follows:

Your number of tokens = （20 – your contribution +（your contribution + Player 2’s contribution) * 1.4）/2）

Please fill in the corresponding amount in the box:

1) If your contribution = **20**: Player 2’s contribution = **20**

Your number of tokens = ☐

2) If your contribution = **0**; Player 2's contribution = **0**

Your number of tokens = ☐

3) If your contribution = **20**; Player 2's contribution = **0**

Your number of tokens= ☐

4) If your contribution = **0**; Player 2's contribution = **20**

Your number of tokens = ☐

3 Experimental Instructions for Experiments 1 and 2

3.1 Experimental Instructions for Experiment 1

3.1.1 Unequal 4020 condition

First of all, thank you so much for joining us. This experiment requires you to play a game with one other person. First you have been assigned an A, which represents your initial number of chips per round. Each chip is equal to a certain percentage of the experimental reward to be purchased for the experimental game. There are ten rounds of the game. You get 40 tokens at the beginning of each round. The other player starts with 20 tokens per round. In the game, you will create a public fund with another player. In each round you have to decide how many tokens to put into the public fund. The other player also needs to make his decision. The total tokens collected into the public fund will have an additional 40% return. Then the new total number of tokens will be divided equally between you. In the next round you will resume the game from the initial 40 tokens. During the game, you need to fill out a record sheet that records how many tokens you put in the public fund in each round, how many tokens another player put in, how many tokens you can recover from the public fund in each round, the number of tokens you have after each round, and the total number of tokens you have after ten rounds. The other player also needs to record the same data. At the end of the game, we will count the number of tokens you have. After the end of the study, the reward of your participation in the experiment will be converted according to a certain percentage, and the specific conversion rules will be determined by the experimenter. Before playing this game, we need to know your current basic emotional state, and the experimenter will give you a scale. I The experiment takes about 30 minutes. If you have any questions about the experiment or if you don't know something about the experiment, please ask the experimenter.

First of all, thank you so much for joining us. This experiment requires you to play a game with one other person. First you have been assigned an A, which represents your initial number of chips per round. Each chip is equal to a certain percentage of the experimental reward to be purchased for the experimental game. There are ten rounds of the game. You get 20 tokens at the beginning of each round. The other player starts with 40 tokens per round. In the game, you will create a public fund with another player. In each round you have to decide how many tokens to put into the public fund. The other player also needs to make his decision. The total tokens collected into the public fund will have an additional 40% return. Then the new total number of tokens will be divided equally between you. In the next round you will resume the game from the initial 40 tokens. During the game, you need to fill out a record sheet that records how many tokens you put in the public fund in each round, how many tokens another player put in, how many tokens you can recover from the public fund in each round, the number of tokens you have

after each round, and the total number of tokens you have after ten rounds. The other player also needs to record the same data. At the end of the game, we will count the number of tokens you have. After the end of the study, the reward of your participation in the experiment will be converted according to a certain percentage, and the specific conversion rules will be determined by the experimenter. Before playing this game, we need to know your current basic emotional state, and the experimenter will give you a scale. I The experiment takes about 30 minutes. If you have any questions about the experiment or if you don't know something about the experiment, please ask the experimenter.

3.1.2 Equal 2020 condition

First of all, thank you so much for joining us. This experiment requires you to play a game with one other person. First you have been assigned B, which represents your initial number of chips per round. Each chip is equal to a certain percentage of the experimental reward to be purchased for the experimental game. There are ten rounds of the game. At the beginning of each round, you and the other player each get 20 chips. In the game, you will create a public fund with another player. In each round you have to decide how many chips to put into the public fund. The other player also needs to make his decision. The total chips collected into the public fund will have an additional 40% return. Then the new total number of chips will be divided equally between you. In the next round you will resume the game from the initial 20 chips. During the game, you need to fill out a record sheet that records how many chips you put in the public fund in each round, how many chips another player put in, how many chips you can recover from the public fund in each round, the number of chips you have after each round, and the total number of chips you have after ten rounds. The other player also needs to record the same data. At the end of the game, we will count the number of chips you have. After the end of the study, the reward of your participation in the experiment will be converted according to a certain percentage, and the specific conversion rules will be determined by the experimenter. Before playing this game, we need to know your current basic emotional state, and the experimenter will give you a scale. The experiment takes about 30 minutes. If you have any questions about the experiment or if you don't know something about the experiment, please ask the experimenter.

Player 1 and 2 read the same public goods game instructions.

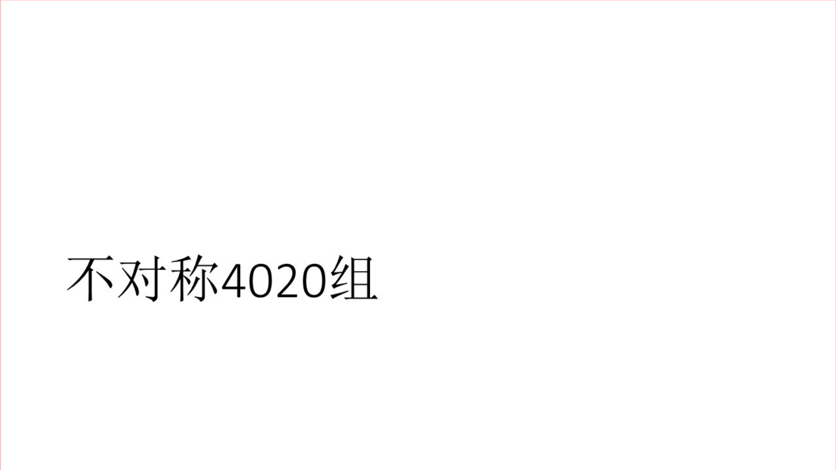

Task: unequal 4020 condition Insert Here

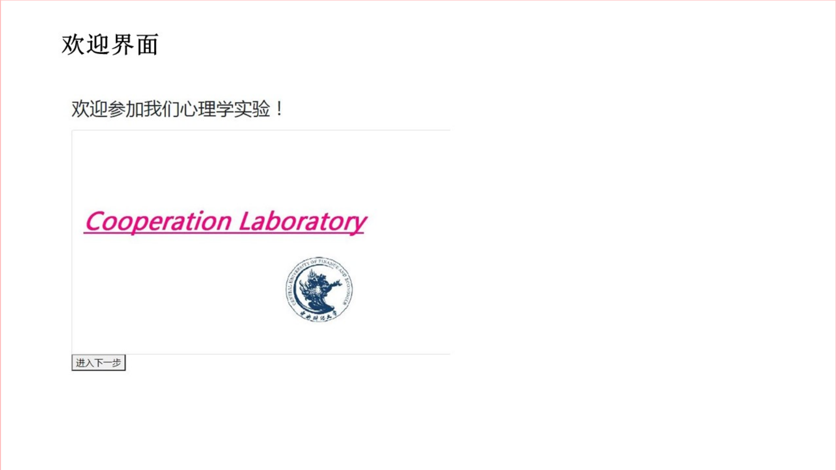

Task:Welcome to the experiment

- 研究内容告知书
    - 研究内容告知书
- 课题:FC001 决策行为的研究
- 本研究已经通过中央财经大学社会与心理学院的研究伦理委员会审查评估。
-
- 在本实验中，你将会与另一个实验参与者共同完成一项决策任务。
- 请注意，实验过程中请不要与另一个位成员沟通。
-
- 以下是你需要完成的任务：
- 1）你需要完成一份调查情绪状态的量表；
- 2）你会与另一名实验参与者共同完成一项决策任务；
- 3）你需要回答一些口头问题。
-
- 如果实验过程中，你有任何问题，请与研究员联系。
-
- 如果你愿意参与或讨论这项研究，请与我们联系。

Task: Research information Research information Topic: Research on FC001 decision behavior This study has been reviewed and evaluated by the Research Ethics Committee of the School of Sociology and Psychology, Central University of Finance and Economics. In this experiment, you will be working with another participant on a decision making task. Please note that you should not communicate with another member during the experiment. Here's what you need to do: 1) You need to complete a scale that examines your emotional state; 2) You will work with another participant on a decision making task; 3) You need to answer some oral questions. If you have any questions during the experiment, please contact the researcher. If you would like to participate in or discuss this study, please contact us.

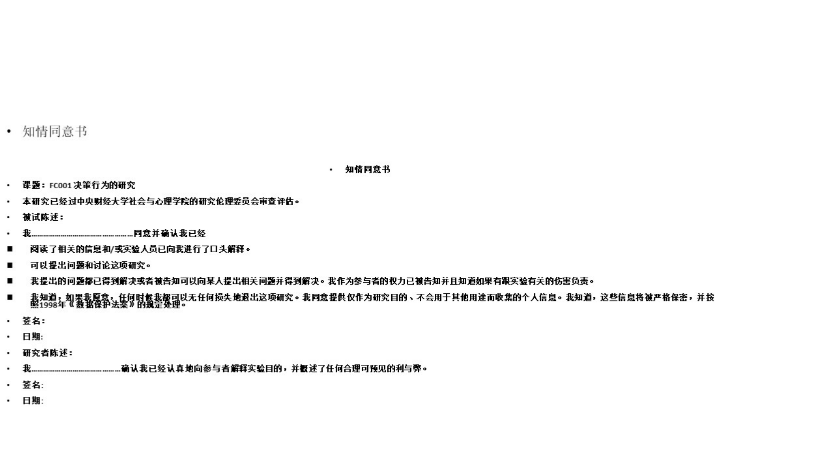

- 知情同意书

- 知情同意书
- 课题：FC001 决策行为的研究
- 本研究已经过中央财经大学社会与心理学院的研究伦理委员会审查评估。
- 被试陈述：
- 我……………………………………同意并确认我已经
  - 阅读了相关的信息和/或实验人员已向我进行了口头解释。
  - 可以提出问题和讨论这项研究。
  - 我提出的问题都已得到解决或者被告知可以向某人提出相关问题并得到解决。我作为参与者的权力已被告知并且知道如果有跟实验有关的伤害负责。
  - 我知道，如果我愿意，任何时候我都可以无任何损失地退出这项研究。我同意提供仅作为研究目的、不会用于其他用途而收集的个人信息。我知道，这些信息将被严格保密，并按照1998年《数据保护法案》的规定处理。
- 签名：
- 日期:
- 研究者陈述：
- 我……………………………………确认我已经认真地向参与者解释实验目的，并概述了任何合理可预见的利与弊。
- 签名:
- 日期:

Task:Informed consent Informed consent Topic: Research on FC001 decision behavior This study has been reviewed and evaluated by the Research Ethics Committee of the School of Sociology and Psychology, Central University of Finance and Economics. Participant statement: I Agree and confirm that I have read the relevant information and/or the experimenter-ed has given me an oral explanation. Questions can be asked and the research discussed. My questions have been addressed or I have been told that I can address the relevant questions to someone and have them addressed. My rights as a participant have been informed and I know if there is any harm associated with the experiment. I knew that I could quit the study at any time with nothing to lose if I wanted to. I agree to provide personal information collected only for research purposes and not for other purposes. I understand that this information will be treated in strict confidence and in accordance with the provisions of the Data Preservation Act 1998. Signature: Date: Researcher statement: I Confirm that I have carefully explained the purpose of the experiment to the participants and outlined any reasonably foreseeable pros and cons. Signature: Date:

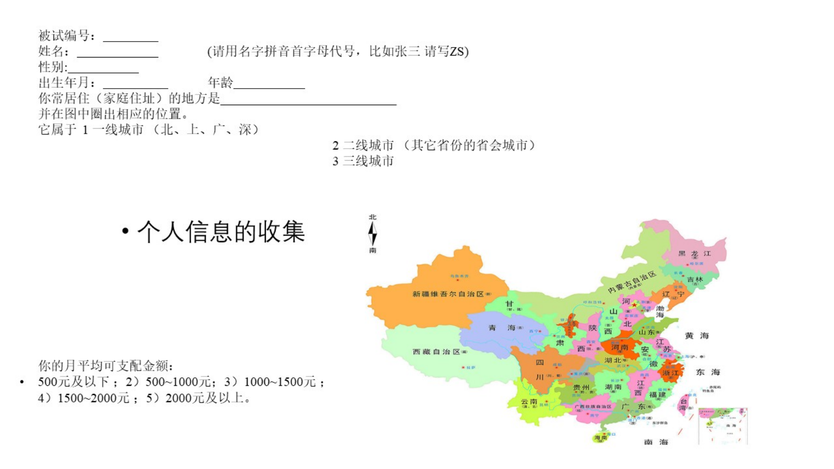

被试编号：________
姓名：___________　　　(请用名字拼音首字母代号，比如张三 请写ZS)
性别:__________
出生年月：__________　　　年龄__________
你常居住（家庭住址）的地方是________________________
并在图中圈出相应的位置。
它属于 1 一线城市（北、上、广、深）
2 二线城市（其它省份的省会城市）
3 三线城市

• 个人信息的收集

你的月平均可支配金额：
• 500元及以下；2）500~1000元；3）1000~1500元；4）1500~2000元；5）2000元及以上。

Task: Collection of personal information

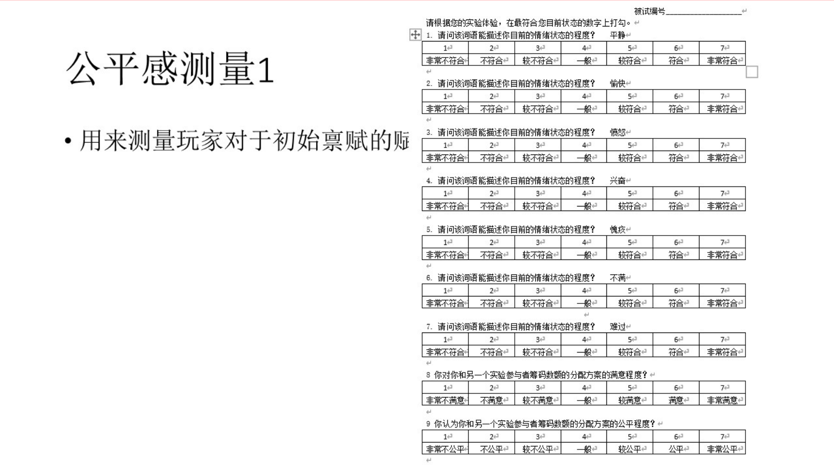

公平感测量1

• 用来测量玩家对于初始禀赋的赈

被试编号__________________

请根据您的实验体验，在最符合您目前状态的数字上打勾。

1. 请问该词语能描述你目前的情绪状态的程度？　平静

1	2	3	4	5	6	7
非常不符合	不符合	较不符合	一般	较符合	符合	非常符合

2. 请问该词语能描述你目前的情绪状态的程度？　愉快

1	2	3	4	5	6	7
非常不符合	不符合	较不符合	一般	较符合	符合	非常符合

3. 请问该词语能描述你目前的情绪状态的程度？　愤怒

1	2	3	4	5	6	7
非常不符合	不符合	较不符合	一般	较符合	符合	非常符合

4. 请问该词语能描述你目前的情绪状态的程度？　兴奋

1	2	3	4	5	6	7
非常不符合	不符合	较不符合	一般	较符合	符合	非常符合

5. 请问该词语能描述你目前的情绪状态的程度？　愧疚

1	2	3	4	5	6	7
非常不符合	不符合	较不符合	一般	较符合	符合	非常符合

6. 请问该词语能描述你目前的情绪状态的程度？　不满

1	2	3	4	5	6	7
非常不符合	不符合	较不符合	一般	较符合	符合	非常符合

7. 请问该词语能描述你目前的情绪状态的程度？　难过

1	2	3	4	5	6	7
非常不符合	不符合	较不符合	一般	较符合	符合	非常符合

8 你对你和另一个实验参与者筹码数额的分配方案的满意程度？

1	2	3	4	5	6	7
非常不满意	不满意	较不满意	一般	较满意	满意	非常满意

9 你认为你和另一个实验参与者筹码数额的分配方案的公平程度？

1	2	3	4	5	6	7
非常不公平	不公平	较不公平	一般	较公平	公平	非常公平

Task:Fairness measurement 1

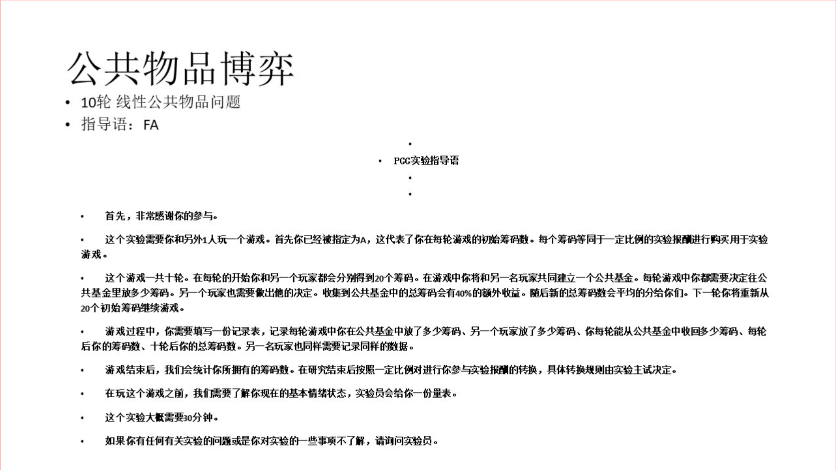

Task:First of all, thank you so much for joining us. This experiment requires you to play a game with one other person. First you have been assigned B, which represents your initial number of chips per round. Each chip is equal to a certain percentage of the experimental reward to be purchased for the experimental game. There are ten rounds of the game. At the beginning of each round, you and the other player each get 20 chips. In the game, you will create a public fund with another player. In each round you have to decide how many chips to put into the public fund. The other player also needs to make his decision. The total chips collected into the public fund will have an additional 40% return. Then the new total number of chips will be divided equally between you. In the next round you will resume the game from the initial 20 chips. During the game, you need to fill out a record sheet that records how many chips you put in the public fund in each round, how many chips another player put in, how many chips you can recover from the public fund in each round, the number of chips you have after each round, and the total number of chips you have after ten rounds. The other player also needs to record the same data. At the end of the game, we will count the number of chips you have. After the end of the study, the reward of your participation in the experiment will be converted according to a certain percentage, and the specific conversion rules will be determined by the experimenter. Before playing this game, we need to know your current basic emotional state, and the experimenter will give you a scale. The experiment takes about 30 minutes. If you have any questions about the experiment or if you don't know something about the experiment, please ask the experimenter.

FB

- PGG实验指导语

- 首先，非常感谢你的参与。
- 这个实验需要你和另外1人玩一个游戏。首先你已经被指定为B，这代表了你每轮游戏的初始筹码数。每个筹码等同于一定比例的实验报酬进行购买用于实验游戏。
- 这个游戏一共十轮。在每轮的开始你和另一个玩家都会分别得到20个筹码。在游戏中你将和另一名玩家共同建立一个公共基金。每轮游戏中你都需要决定在公共基金里放多少筹码。另一个玩家也需要做出他的决定。收集到公共基金中的总筹码会有40%的额外收益。随后新的总筹码数会平均的分给你们。下一轮你将重新从20个初始筹码继续游戏。
- 游戏过程中，你需要填写一份记录表，记录每轮游戏中你在公共基金中放了多少筹码、另一个玩家放了多少筹码、你每轮能从公共基金中收回多少筹码、每轮后你的筹码数、十轮后你的总筹码数。另一名玩家也同样需要记录同样的数据。
- 游戏结束后，我们会统计你所拥有的筹码数。在研究结束后按照一定比例对进行你参与实验报酬的转换，具体转换规则由实验主试决定。
- 在玩这个游戏之前，我们需要了解你现在的基本情绪状态，实验员会给你一份量表。
- 这个实验大概需要30分钟。
- 如果你有任何有关实验的问题或是你对实验的一些事项不了解，请询问实验员。

Task: First of all, thank you so much for joining us. This experiment requires you to play a game with one other person. First you have been assigned B, which represents your initial number of chips per round. Each chip is equal to a certain percentage of the experimental reward to be purchased for the experimental game. There are ten rounds of the game. At the beginning of each round, you and the other player each get 20 chips. In the game, you will create a public fund with another player. In each round you have to decide how many chips to put into the public fund. The other player also needs to make his decision. The total chips collected into the public fund will have an additional 40% return. Then the new total number of chips will be divided equally between you. In the next round you will resume the game from the initial 20 chips. During the game, you need to fill out a record sheet that records how many chips you put in the public fund in each round, how many chips another player put in, how many chips you can recover from the public fund in each round, the number of chips you have after each round, and the total number of chips you have after ten rounds. The other player also needs to record the same data. At the end of the game, we will count the number of chips you have. After the end of the study, the reward of your participation in the experiment will be converted according to a certain percentage, and the specific conversion rules will be determined by the experimenter. Before playing this game, we need to know your current basic emotional state, and the experimenter will give you a scale. The experiment takes about 30 minutes. If you have any questions about the experiment or if you don't know something about the experiment, please ask the experimenter.

UFA

- PGG实验指导语

- 首先，非常感谢你的参与。
- 这个实验需要你和另外1人玩一个游戏。首先你已经被指定为A，这代表了你每轮游戏的初始筹码数。每个筹码等同于一定比例的实验报酬进行购买用于实验游戏。
- 这个游戏一共十轮。在每轮的开始你都会得到40个筹码。而另一个玩家则是每轮都会有20个初始筹码。在游戏中你将和另一名玩家共同建立一个公共基金。每轮游戏中你都需要决定往公共基金里放多少筹码。另一个玩家也需要做出他的决定。收集到公共基金中的总筹码会有40%的额外收益。随后新的总筹码数会平均的分给你们。下一轮你将重新从40个初始筹码继续游戏。
- 游戏过程中，你需要填写一份记录表，记录每轮游戏中你在公共基金中放了多少筹码、另一个玩家放了多少筹码、你每轮能从公共基金中收回多少筹码、每轮后你的筹码数、十轮后你的总筹码数。另一名玩家也同样需要记录同样的数据。
- 游戏结束后，我们会统计你所拥有的筹码数。在研究结束后按照一定比例对进行你参与实验报酬的转换，具体转换规则由实验主试决定。
- 在玩这个游戏之前，我们需要了解你现在的基本情绪状态，实验员会给你一份量表。
- 这个实验大概需要30分钟。
- 如果你有任何有关实验的问题或是你对实验的一些事项不了解，请询问实验员。

Task: First of all, thank you so much for joining us. This experiment requires you to play a game with one other person. First you have been assigned an A, which represents your initial number of chips per round. Each chip is equal to a certain percentage of the experimental reward to be purchased for the experimental game. There are ten rounds of the game. You get 40 tokens at the beginning of each round. The other player starts with 20 tokens per round. In the game, you will create a public fund with another player. In each round you have to decide how many tokens to put into the public fund. The other player also needs to make his decision. The total tokens collected into the public fund will have an additional 40% return. Then the new total number of tokens will be divided equally between you. In the next round you will resume the game from the initial 40 tokens. During the game, you need to fill out a record sheet that records how many tokens you put in the public fund in each round, how many tokens another player put in, how many tokens you can recover from the public fund in each round, the number of tokens you have after each round, and the total number of tokens you have after ten rounds. The other player also needs to record the same data. At the end of the game, we will count the number of tokens you have. After the end of the study, the reward of your participation in the experiment will be converted according to a certain percentage, and the specific conversion rules will be determined by the experimenter. Before playing this game, we need to know your current basic emotional state, and the experimenter will give you a scale. I The experiment takes about 30 minutes. If you have any questions about the experiment or if you don't know something about the experiment, please ask the experimenter.

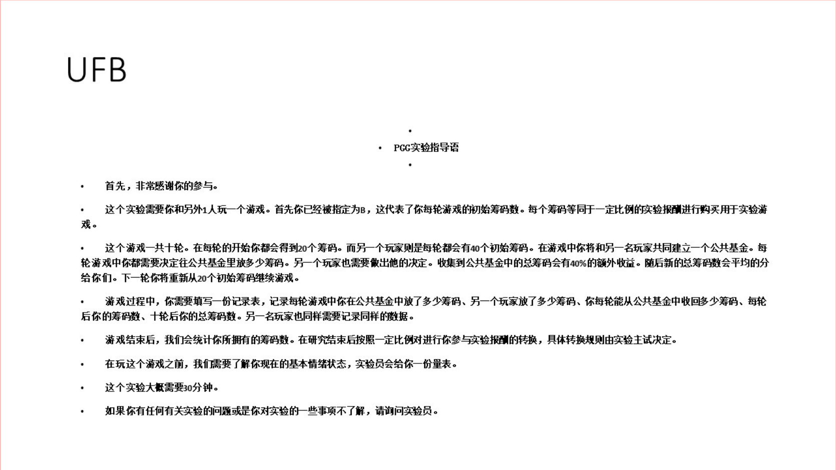

UFB

- PGG实验指导语

- 首先，非常感谢你的参与。
- 这个实验需要你和另外1人玩一个游戏。首先你已经被指定为B，这代表了你每轮游戏的初始筹码数。每个筹码等同于一定比例的实验报酬进行购买用于实验游戏。
- 这个游戏一共十轮。在每轮的开始你都会得到20个筹码。而另一个玩家则是每轮都会有40个初始筹码。在游戏中你将和另一名玩家共同建立一个公共基金。每轮游戏中你都需要决定往公共基金里放多少筹码。另一个玩家也需要做出他的决定。收集到公共基金中的总筹码会有40%的额外收益。随后新的总筹码数会平均的分给你们。下一轮你将重新从20个初始筹码继续游戏。
- 游戏过程中，你需要填写一份记录表，记录每轮游戏中你在公共基金中放了多少筹码、另一个玩家放了多少筹码、你每轮能从公共基金中收回多少筹码、每轮后你的筹码数、十轮后你的总筹码数。另一名玩家也同样需要记录同样的数据。
- 游戏结束后，我们会统计你所拥有的筹码数。在研究结束后按照一定比例对进行你参与实验报酬的转换，具体转换规则由实验主试决定。
- 在玩这个游戏之前，我们需要了解你现在的基本情绪状态，实验员会给你一份量表。
- 这个实验大概需要30分钟。
- 如果你有任何有关实验的问题或是你对实验的一些事项不了解，请询问实验员。

Task: First of all, thank you so much for joining us. This experiment requires you to play a game with one other person. First you have been assigned an A, which represents your initial number of chips per round. Each chip is equal to a certain percentage of the experimental reward to be purchased for the experimental game. There are ten rounds of the game. You get 20 tokens at the beginning of each round. The other player starts with 40 tokens per round. In the game, you will create a public fund with another player. In each round you have to decide how many tokens to put into the public fund. The other player also needs to make his decision. The total tokens collected into the public fund will have an additional 40% return. Then the new total number of tokens will be divided equally between you. In the next round you will resume the game from the initial 40 tokens. During the game, you need to fill out a record sheet that records how many tokens you put in the public fund in each round, how many tokens another player put in, how many tokens you can recover from the public fund in each round, the number of tokens you have after each round, and the total number of tokens you have after ten rounds. The other player also needs to record the same data. At the end of the game, we will count the number of tokens you have. After the end of the study, the reward of your participation in the experiment will be converted according to a certain percentage, and the specific conversion rules will be determined by the experimenter. Before playing this game, we need to know your current basic emotional state, and the experimenter will give you a scale. I The experiment takes about 30 minutes. If you have any questions about the experiment or if you don't know something about the experiment, please ask the experimenter.

完成10轮的线性公共物品

Task:Complete 10 rounds of linear public goods game

公平感测量2

被试编号

请根据您的实际体验，在最符合您目前状态的数字上打勾。

1. 请问该词语能描述你目前的情绪状态的程度？ 平静

1	2	3	4	5	6	7
非常不符合	不符合	较不符合	一般	较符合	符合	非常符合

2. 请问该词语能描述你目前的情绪状态的程度？ 愉快

1	2	3	4	5	6	7
非常不符合	不符合	较不符合	一般	较符合	符合	非常符合

3. 请问该词语能描述你目前的情绪状态的程度？ 愤怒

1	2	3	4	5	6	7
非常不符合	不符合	较不符合	一般	较符合	符合	非常符合

4. 请问该词语能描述你目前的情绪状态的程度？ 兴奋

1	2	3	4	5	6	7
非常不符合	不符合	较不符合	一般	较符合	符合	非常符合

5. 请问该词语能描述你目前的情绪状态的程度？ 愧疚

1	2	3	4	5	6	7
非常不符合	不符合	较不符合	一般	较符合	符合	非常符合

6. 请问该词语能描述你目前的情绪状态的程度？ 不满

1	2	3	4	5	6	7
非常不符合	不符合	较不符合	一般	较符合	符合	非常符合

7. 请问该词语能描述你目前的情绪状态的程度？ 难过

1	2	3	4	5	6	7
非常不符合	不符合	较不符合	一般	较符合	符合	非常符合

8. 你对你和另一个实验参与者筹码数额的分配分配方案的满意程度？

1	2	3	4	5	6	7
非常不满意	不满意	较不满意	一般	较满意	满意	非常满意

9. 你认为你和另一个实验参与者筹码数额的分配的公平程度？

1	2	3	4	5	6	7
非常不公平	不公平	较不公平	一般	较公平	公平	非常公平

Task:Fairness measurement 2

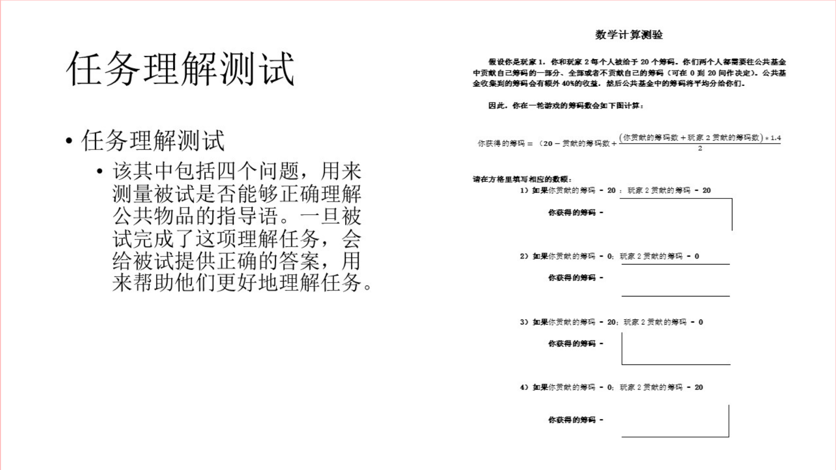

Task:Task understanding test It consisted of four questions designed to measure whether participants could correctly understand instructions for public goods. Once the participants completed the comprehension task, they were given the correct answers to help them understand the task better

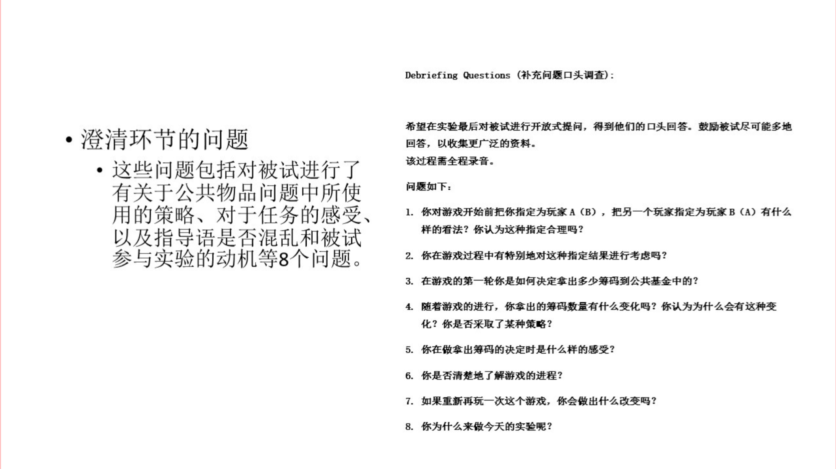

Task: Clarify the links These questions included eight questions about the strategy used in the public goods question, the feelings about the task, whether the instruction was confused and the motivation of the subjects to participate in the experiment.

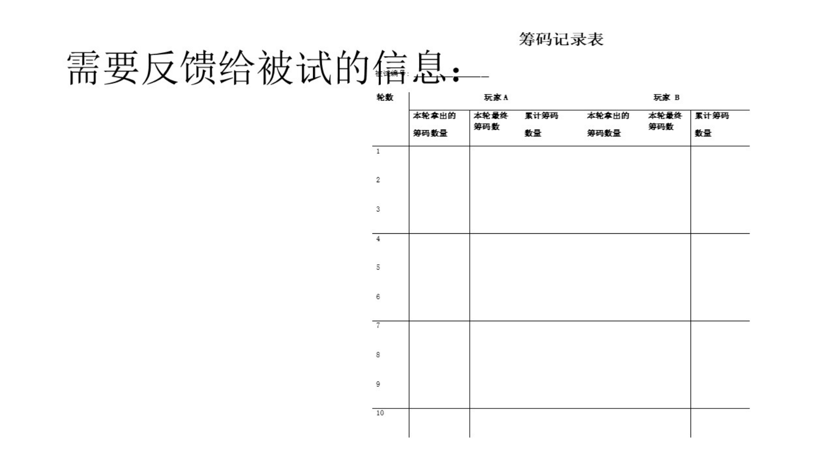

轮数	玩家 A			玩家 B		
	本轮拿出的筹码数量	本轮最终筹码数	累计筹码数量	本轮拿出的筹码数量	本轮最终筹码数	累计筹码数量
1						
2						
3						
4						
5						
6						
7						
8						
9						
10						

Task: Information that needs to be fed back to the participants

3.1.3 Equal2020 condition

The instruction in the Equal2020 condition were idential with the Unequal4020 condition except players were assigned with equal endowments in the begining of the games rather than unequal endowments.

3.2 Experimental Instructions for Experiment 2

3.2.1 Unequal3010 condition

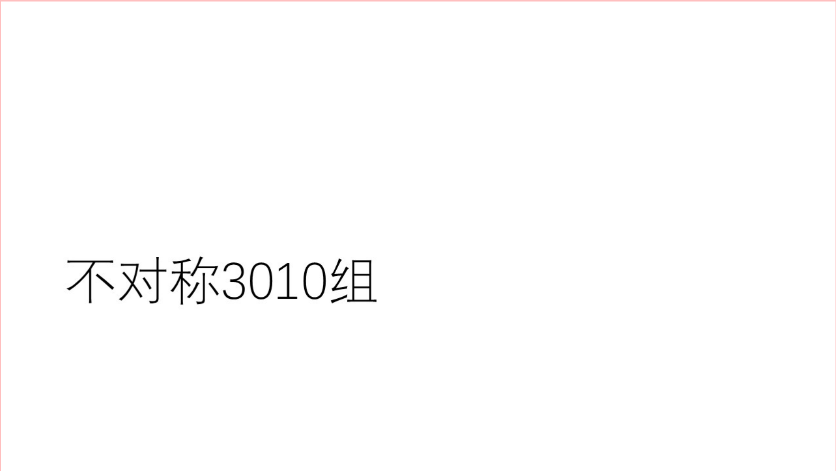

Task: Unequall 3010 condition

欢迎界面

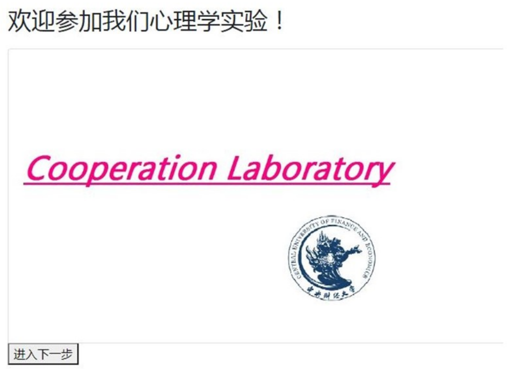

Task:Welcome to the experiment

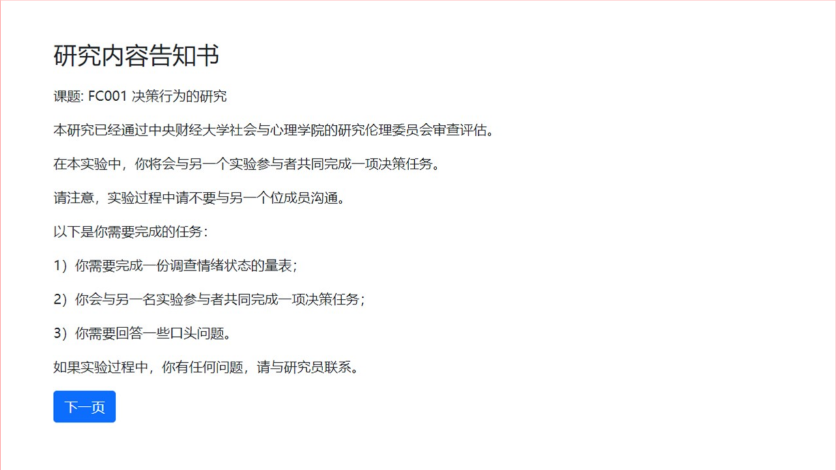

研究内容告知书

课题: FC001 决策行为的研究

本研究已经通过中央财经大学社会与心理学院的研究伦理委员会审查评估。

在本实验中，你将会与另一个实验参与者共同完成一项决策任务。

请注意，实验过程中请不要与另一个位成员沟通。

以下是你需要完成的任务：

1）你需要完成一份调查情绪状态的量表；

2）你会与另一名实验参与者共同完成一项决策任务；

3）你需要回答一些口头问题。

如果实验过程中，你有任何问题，请与研究员联系。

下一页

Task: Research information Topic: Research on FC001 decision behavior This study has been reviewed and evaluated by the Research Ethics Committee of the School of Sociology and Psychology, Central University of Finance and Economics. In this experiment, you will be working with another participant on a decision making task. Please note that you should not communicate with another member during the experiment. Here's what you need to do 1) You need to complete a scale that examines your emotional state; 2) You will work with another participant on a decision making task; 3) You need to answer some oral questions. If you have any questions during the experiment, please contact the researcher,

知情同意书

课题：FC001 决策行为的研究 本研究已经通过中央财经大学社会与心理学院的研究伦理委员会审查评估。

我————————————————————————————同意并确认我已经：

1 阅读了相关的信息和/或实验人员已向我进行了口头解释。

2 可以提出问题和讨论这项研究。

3. 提出的问题都已经得到解决或被告知可以向某人提出相关问题并得到解决。我作为参与者的权力已被告知并且知道如果有跟实验相关的权利。

4. 我知道，如果我愿意，任何时候我都可以无任何损失地退出这项研究。我同意提供仅作为研究目的、不会用于其他用途而收集的个人信息。

我知道这些信息将会被严格保密，并根据相关法律进行处理。

[我愿意参加此实验并已知晓相关事宜，点击此处，进入下一步]

Task: Informed consent Topic: Research on FC001 Decision Behavior This study has been reviewed by the Research Ethics Committee of the School of Sociology and Psychology, Central University of Finance and Economics. I agree and confirm that I have: 1 Read the relevant information and/or the experimentered has orally explained it to me. 2 You can ask questions and discuss the research. 3. The issues raised have been addressed or have been informed that the relevant issues can be addressed to someone and resolved. My rights as a participant have been Inform and know if there are rights related to the experiment. 4. I knew I could quit the study at any time with nothing to lose if I wanted to. I agree to provide it for research purposes only. Personal information collected that will not be used for other purposes. I understand that this information will be treated in strict confidence and handled in accordance with relevant laws.

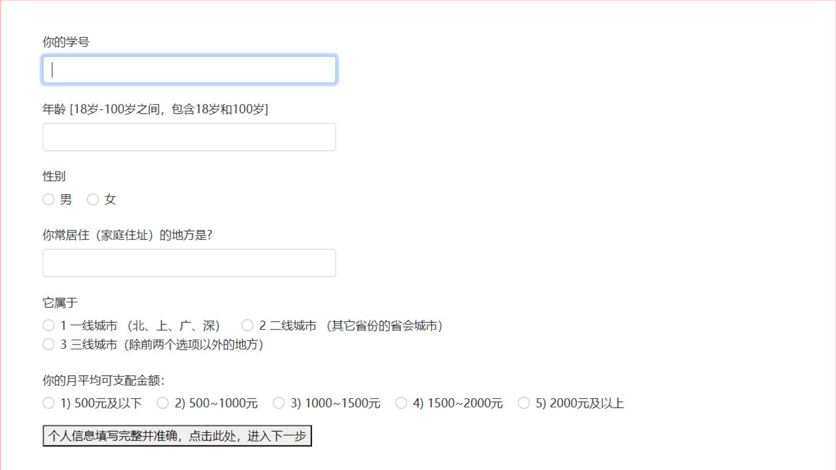

Task: Collection of personal information

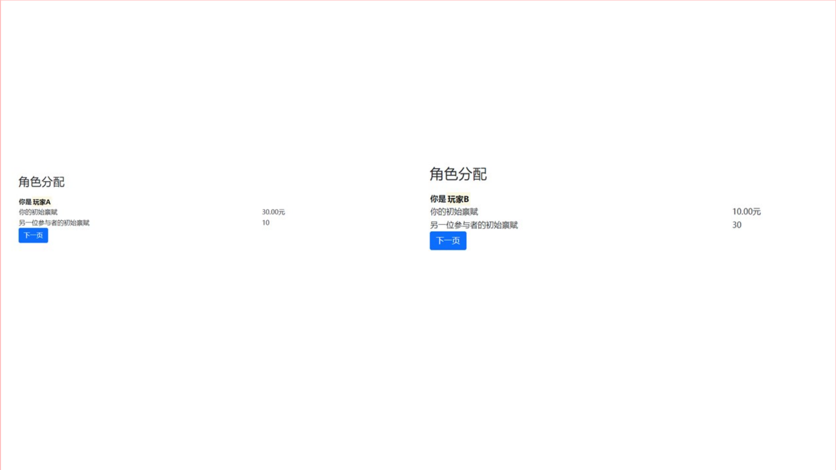

Task: Role assignment Show the starting talent of yourself and the opposing player.

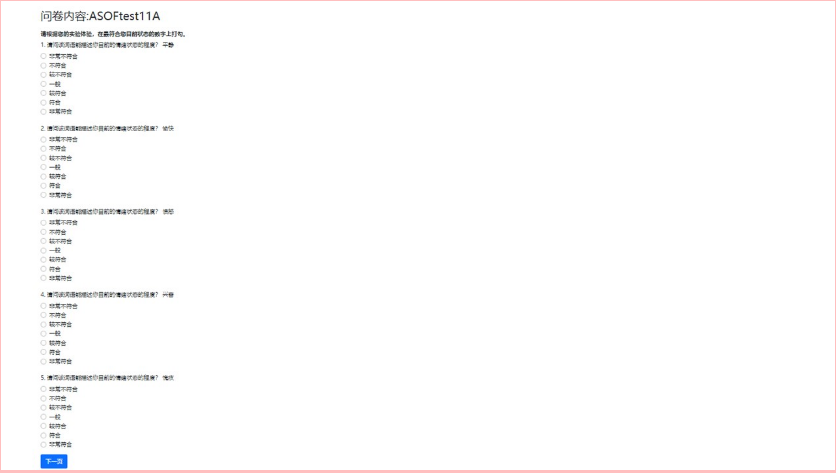

问卷内容:ASOFtest11A

请根据您的实验体验，在最符合您目前状态的数字上打勾。

1. 请问该词语能描述你目前的情绪状态的程度？ 平静
- ○ 非常不符合
- ○ 不符合
- ○ 较不符合
- ○ 一般
- ○ 较符合
- ○ 符合
- ○ 非常符合

2. 请问该词语能描述你目前的情绪状态的程度？ 愉快
- ○ 非常不符合
- ○ 不符合
- ○ 较不符合
- ○ 一般
- ○ 较符合
- ○ 符合
- ○ 非常符合

3. 请问该词语能描述你目前的情绪状态的程度？ 愤怒
- ○ 非常不符合
- ○ 不符合
- ○ 较不符合
- ○ 一般
- ○ 较符合
- ○ 符合
- ○ 非常符合

4. 请问该词语能描述你目前的情绪状态的程度？ 兴奋
- ○ 非常不符合
- ○ 不符合
- ○ 较不符合
- ○ 一般
- ○ 较符合
- ○ 符合
- ○ 非常符合

5. 请问该词语能描述你目前的情绪状态的程度？ 愧疚
- ○ 非常不符合
- ○ 不符合
- ○ 较不符合
- ○ 一般
- ○ 较符合
- ○ 符合
- ○ 非常符合

下一页

Task:Fill in the questionnaire. Questionnaire content: ASOFtest11A.

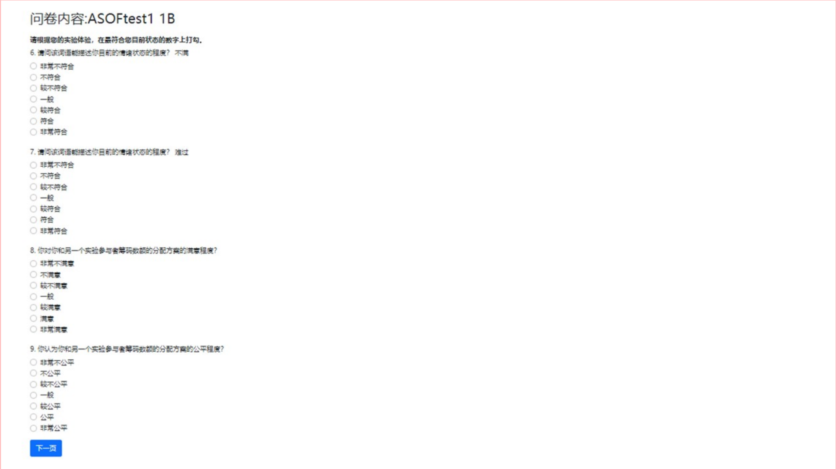

问卷内容:ASOFtest1 1B

请根据您的实验体验，在最符合您目前状态的数字上打勾。

6. 请问该词语能描述你目前的情绪状态的程度？ 不满
- ○ 非常不符合
- ○ 不符合
- ○ 较不符合
- ○ 一般
- ○ 较符合
- ○ 符合
- ○ 非常符合

7. 请问该词语能描述你目前的情绪状态的程度？ 难过
- ○ 非常不符合
- ○ 不符合
- ○ 较不符合
- ○ 一般
- ○ 较符合
- ○ 符合
- ○ 非常符合

8. 你对你和另一个实验参与者筹码数额的分配方案的满意程度？
- ○ 非常不满意
- ○ 不满意
- ○ 较不满意
- ○ 一般
- ○ 较满意
- ○ 满意
- ○ 非常满意

9. 你认为你和另一个实验参与者筹码数额的分配方案的公平程度？
- ○ 非常不公平
- ○ 不公平
- ○ 较不公平
- ○ 一般
- ○ 较公平
- ○ 公平
- ○ 非常公平

下一页

Task: Fill in the questionnaire. Questionnaire content: ASOFtest11B.

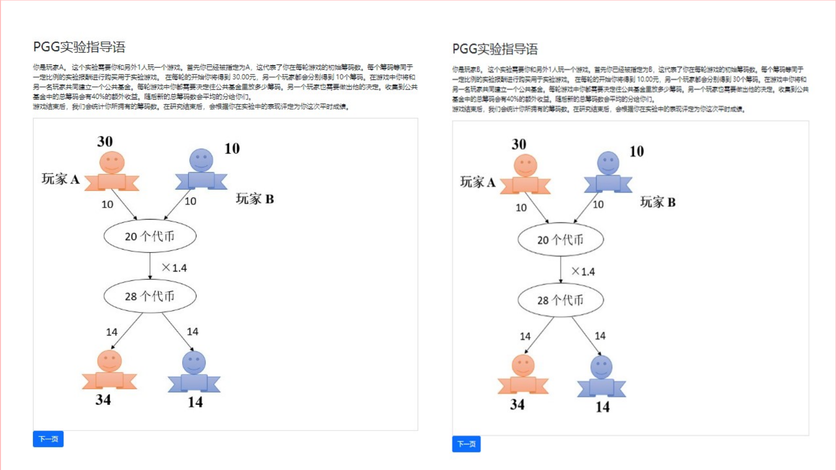

Task: PGG experimental guidelines.You are player A/B, and this experiment requires you to play A game with another person. First, you have been designated as A, which represents your initial tokens in each round of the game, and each token is equal to a certain percentage of the experimental reward to buy for the experimental game. At the beginning of each round you will get XXX tokens, another player will get XXX tokens, in the game you will build a public fund with another player, in each round you need to decide how many tokens to put in the public fund, the other player also needs to make his decision, The total tokens collected from the public fund will have an additional return of 40%, and then the new total tokens will be evenly distributed to you. After the game is over, we will count the tokens you have, and at the end of the study, we will evaluate your average score according to your performance in the experiment.

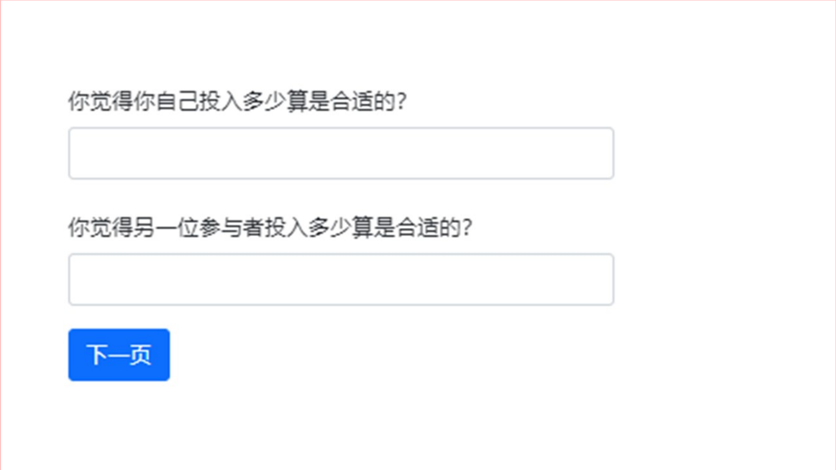

Task: How much of yourself do you think is appropriate? How much input do you think is appropriate for the other participant?

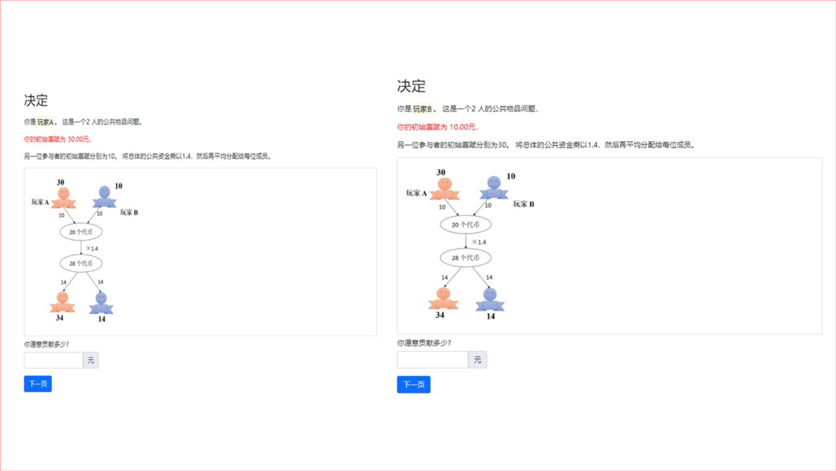

Task:Decide that you are player A/B.This is a two-person public goods problem.Your initial endowment of XXX, the initial contribution of another participant of XXX multiplies the total public funds by 1.4, and then distributes them equally to each member.

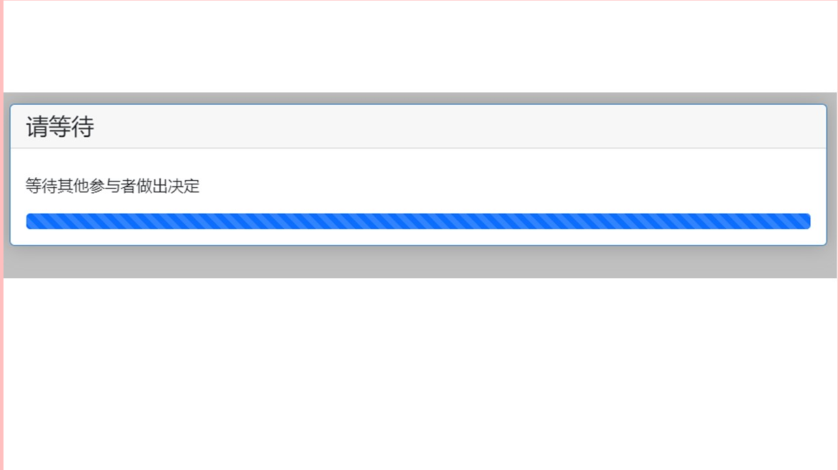

Task: Wait for the other participants to decide.

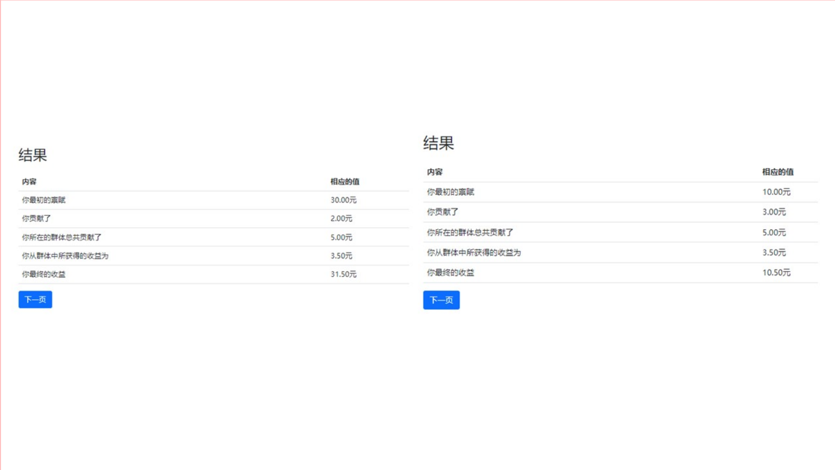

Task: Result feedback.Your original endowment.You contributed.Your group has contributed in total.The payoff you get from the group is.Your final profit.

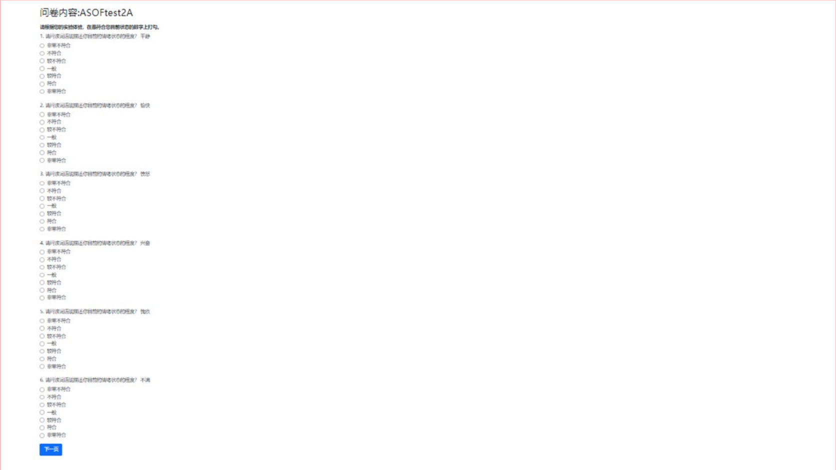

Task: Fill in the questionnaire.Questionnaire content: ASOFtest2A.

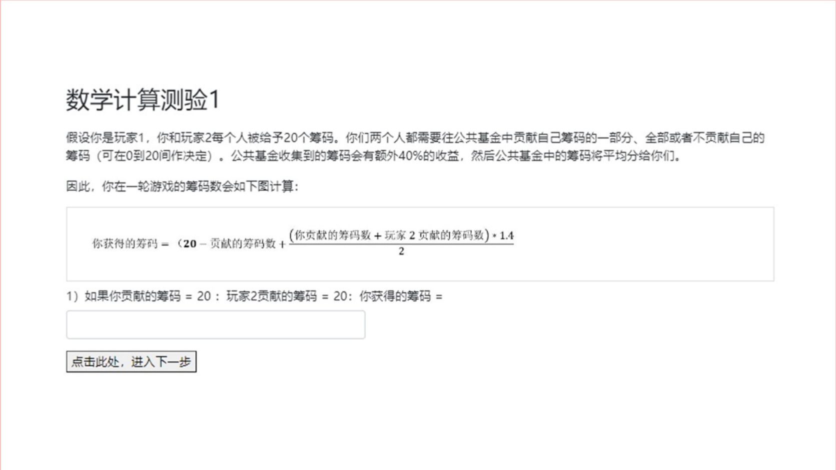

Task:Mathematical calculation test 1.Suppose you are Player 1 and you and player 2 are each given 20 tokens, and both of you are required to contribute some, all, or none of your tokens to the public fund (on a scale of 0 to 20). The chips collected by the public fund will receive an additional 40%, and the chips from the public fund will be divided equally among you. Therefore, the number of tokens you have in a round is calculated as follows: You gain leverage = (20 The number of chips contributed +(The number of chips you contributed + The number of chips contributed by Player 2) 1.4)/2).1) If you contributed tokens =20: Player 2 contributed tokens =20: Tokens you earned =?

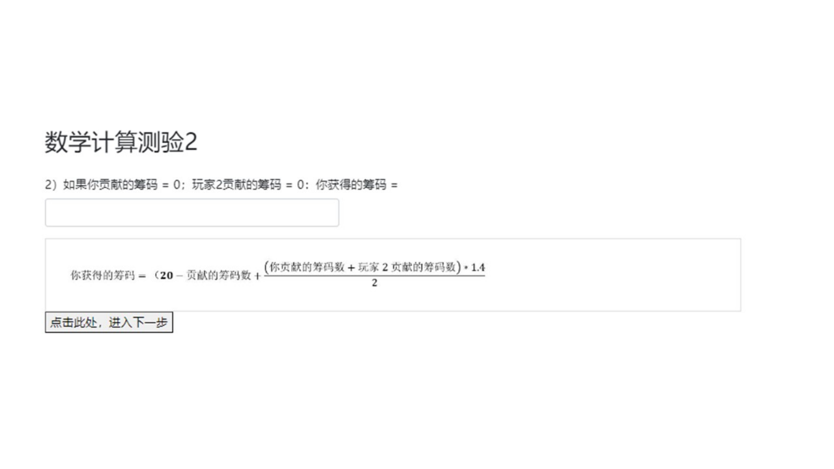

Task: Mathematical calculation Test 2.2) If you contributed tokens =0: Player 2 contributed tokens =0: Tokens you earned =? You gain leverage = (20 The number of chips contributed +(The number of chips you contributed + The number of chips contributed by Player 2) 1.4)/2).

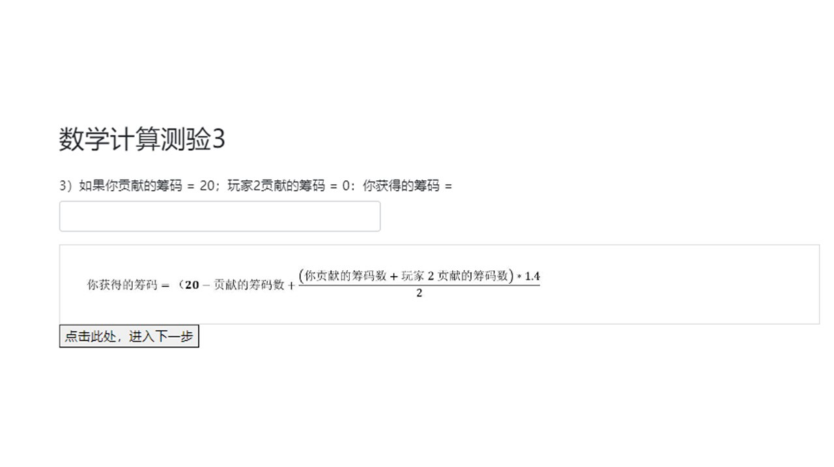

Task: Mathematical calculation Test 2.2) If you contributed tokens =20: Player 2 contributed tokens =0: Tokens you earned =? You gain leverage = (20 The number of chips contributed +(The number of chips you contributed + The number of chips contributed by Player 2) 1.4)/2).

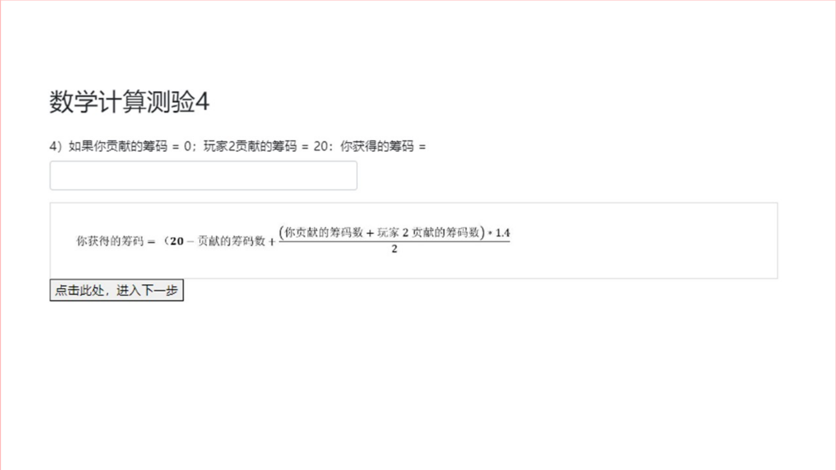

Task: Mathematical calculation Test 4.4) If you contributed tokens =0: Player 2 contributed tokens =20: Tokens you earned =? You gain leverage = (20 The number of chips contributed +(The number of chips you contributed + The number of chips contributed by Player 2) 1.4)/2).

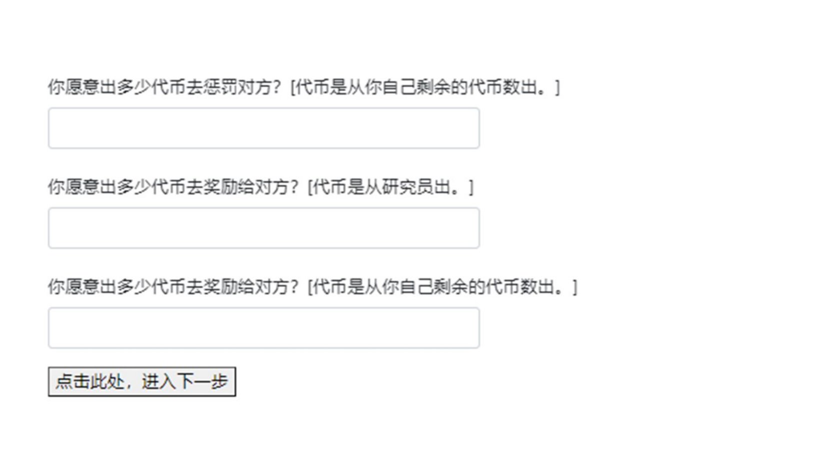

Task: How many tokens are you willing to pay to punish the other party? (Tokens are counted from your own remaining tokens.) How many tokens are you willing to give to the other party? (Tokens are issued from the researcher.) How many tokens are you willing to give to the other party? (Tokens are counted from your own remaining tokens.)

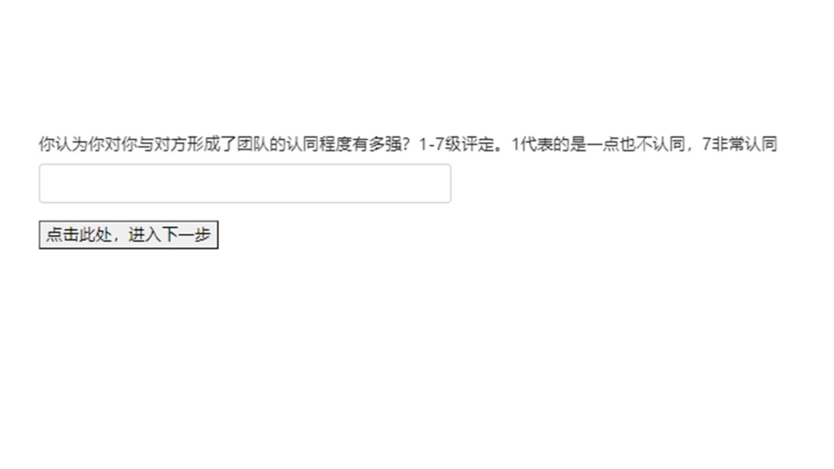

Task: How strongly do you think you identify with each other as a team? Scale 1-7. One means not at all, and seven means very much.

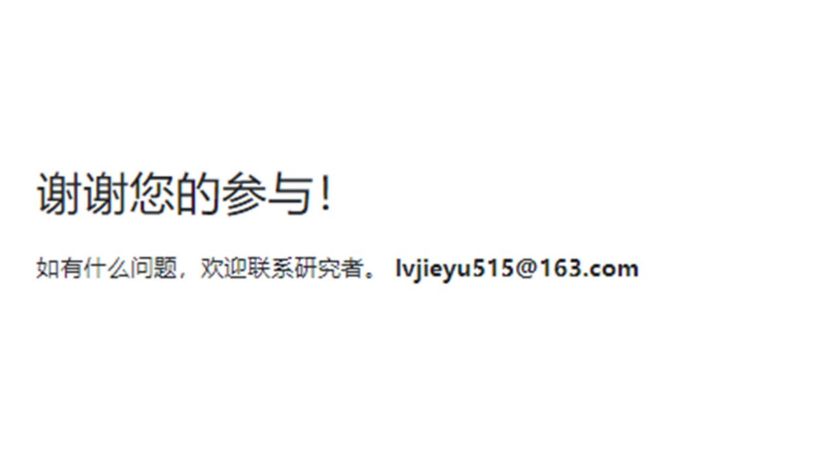

Task: Thank you for your participation

3.2.2 Unequal4020 condition

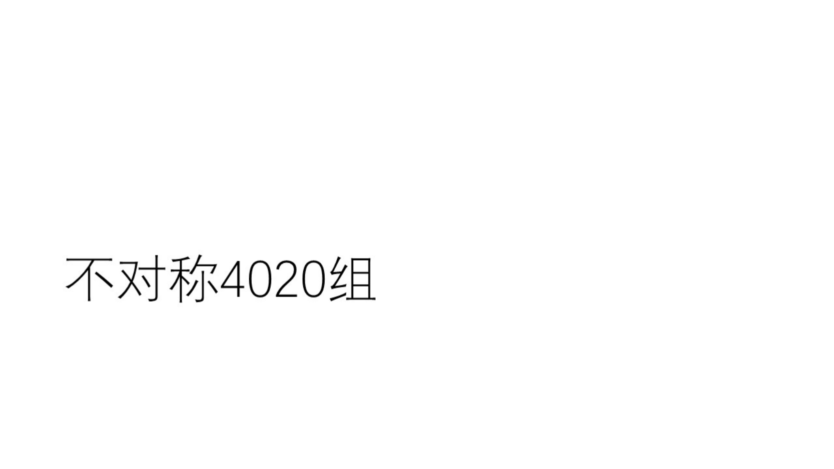

Task:Unequal 4020 condition

Task:

3.2.3 Equal 2020 condition

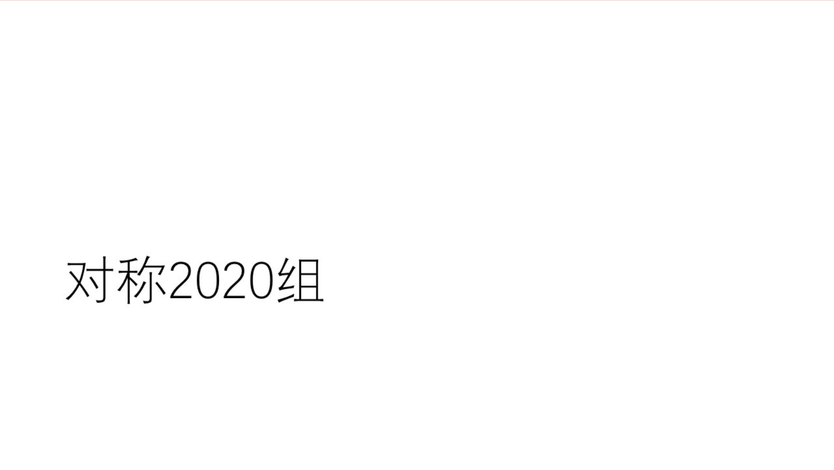

Task: Equal 2020 condition

Task:
